# Historical ethnopharmacology of the herbalists from Krummhübel in the Sudety Mountains (seventeenth to nineteenth century), Silesia

**DOI:** 10.1186/s13002-019-0298-z

**Published:** 2019-05-23

**Authors:** Krzysztof Spałek, Izabela Spielvogel, Małgorzata Proćków, Jarosław Proćków

**Affiliations:** 1 Division of Botany, Institute of Biology, University of Opole, Oleska 22, 45-052 Opole, Poland; 2 Department of Physiotherapy, Institute of Physiotherapy, Opole University of Technology, Prószkowska 76, 45-758 Opole, Poland; 3 Museum of Natural History, University of Wrocław, Sienkiewicza 21, 50-335 Wrocław, Poland; 4 Department of Plant Biology, Institute of Biology, Faculty of Biology and Animal Science, Wrocław University of Environmental and Life Sciences, Kożuchowska 5b, 51-631 Wrocław, Poland

**Keywords:** Medicinal plants, Mixtures, Folk medicine, Phytotherapy history, Phytopharmacy, Ethnobotany

## Abstract

**Background:**

Krummhübel (after 1945, Karpacz) in the Sudety Mountains (now SW Poland) was called “the village of pharmacists”. At the end of the seventeenth century, there were 57 households, of which about 40 were inhabited by herbalists. Krummhübel herbalists were the first in the Sudety region who applied medicinal mixtures for the treatment of various diseases (using, among others, plants, oils, minerals and even viper venom) in contrast to previous herbalists who only indicated the use of individual plant species for specific diseases. Riesengebirge (in Polish Karkonosze) potions were sold in Austria, the Czech Republic, Poland and Russia, and some of them could even be purchased in Scandinavia and England. The purpose of this paper is an ethnopharmacological analysis of historical texts of herbalists from Krummhübel. Based on their recipes, we analysed the use reports of drugs. Recently, research on ethnobotany and ethnopharmacological analyses of historical materials or egodocuments related to formulations used in folk medicine have become an important source of acquiring knowledge about new medicines.

**Methods:**

Based on 46 recipes of Krummhübel herbalists re-written by Reitzig (1943), we analysed the use reports of drugs which included plant taxa and other constituents such as animal formulations, fungi, inorganic and organic substances and minerals as well as tinctures (with alcohol/spirit) and elixirs (without alcohol/spirit). For each usage mentioned in the text, we recorded (i) the putative botanical identity of the taxon; (ii) the plant family or origin of other than the plant constituent; (iii) the reported plant part; (iv) the number of the recipe; (v) the name of the recipe; (vi) the vernacular name of ingredient; (vii) the described symptom, ailment or specific use; (viii) our modern (viz. biomedical) interpretation of the described symptom or ailment; (ix) the mode of administration; and (x) the category of use under which we filed the specific use. We also cross-checked the medicinal plants of Krummhübel herbalists with the species described in old manuscripts and regional surveys and compared their use with contemporary plant use.

**Results:**

The paper introduces the generated database comprising 348 use reports of 46 drugs based on 70 plant taxa and other constituents. Besides, we address patterns such as the frequent recommendation of Fabaceae herbs for respiratory system issue and gynaecology and Asteraceae for respiratory system and cardiovascular problems. Gastrointestinal use reports are based on Asphodelaceae, Burseraceae and Rosaceae species.

**Conclusions:**

Remedies that lost importance over time as well as drugs used for diseases now controlled by conventional medicine may be interesting starting points for research on herbal medicine and drug discovery. It seems to be important to attempt to reproduce therapeutic mixtures from the preserved recipes of Krummhübel herbalists, which offers an opportunity to learn more about the real effects of the former medicines and their therapeutic activity. The obtained data can also be used to search for new drugs.

**Electronic supplementary material:**

The online version of this article (10.1186/s13002-019-0298-z) contains supplementary material, which is available to authorized users.

## Background

Caspar Schwenckfeld, a municipal doctor from Hirschberg (after 1945, Jelenia Góra), was one of the first medicinal plant explorers operating in the Silesia region. Two monographs of great value are the result of his scientific work. Chronologically, his scientific work describing resorts in Warmbrunn (after 1945, Cieplice), Landeck (after 1945, Lądek-Zdrój), Flinsberg (after 1945, Świeradów) and Salzbrunn (after 1945, Szczawno) appeared first [1, 2], and 7 years later, another monograph was released. It describes 50 species of plants that were used in phytotherapy at the turn of the seventeenth century in health resorts in the Sudety Mountains (in German, Sudeten), SW Poland, mainly in Warmbrunn [3]. Many species of medicinal plants formerly used in phytopharmacy now have scientifically demonstrated medicinal properties based on their diverse chemical compositions (e.g. [4–8]).

The purpose of the work is an ethnopharmacological analysis of historical texts of the so-called Krummhübel laboratory workers, who were active in the Sudetes (Central Europe) from the seventeenth to nineteenth centuries. Our research will contribute to a better understanding of treatments for diseases in this region prior to the development of the pharmacological industry. It will also provide in-depth insight into old methods of treatment. So far, no research has been carried out on ethnopharmacological activities of the herbalists from Krummhübel. In particular, this study may guide research on novel phyto-therapeutic agents, inform safety evaluations and help to prove the tradition of use in terms of drug regulations [9, 10].

We also cross-checked the medicinal plants of Krummhübel herbalists with the species described in five manuscripts and regional surveys, including Matthioli (1563) [11], Schwenckfeld (1607) [3], Mattuschka (1779) [12], Kneipp (1892) [13], Fischer (1930s) [14] and Madaus (1938) [15]. Besides, we compared their uses with those of other contemporary plants.

Recently, research on ethnobotany and ethnopharmacological analysis of historical materials or egodocuments, related to preparations used in folk medicine, has become an important source of acquiring knowledge about new medicines [10, 16]. Our study may constitute a part of this trend.

Karpacz (formerly Krummhübel) is a town located in Silesia in Poland. Until the mid-sixteenth century, this region belonged to the Kingdom of Bohemia, and in 1526, it became a part of the Habsburg Empire. As a result of the Silesian wars in the years 1740–1742, Silesia came under the rule of the Kingdom of Prussia and remained within the German borders until 1945. After World War II, under the terms of the agreements at the Yalta Conference and the Potsdam Agreement (both in 1945), German Silesia, east of the rivers Oder (now Odra) and Lusatian Neisse (now Nysa Łużycka), was transferred to Poland. This region included Krummhübel (after 1945 renamed Karpacz), the area of the herbalists’ activities described in this article [17, 18].

**Fig. 1:**
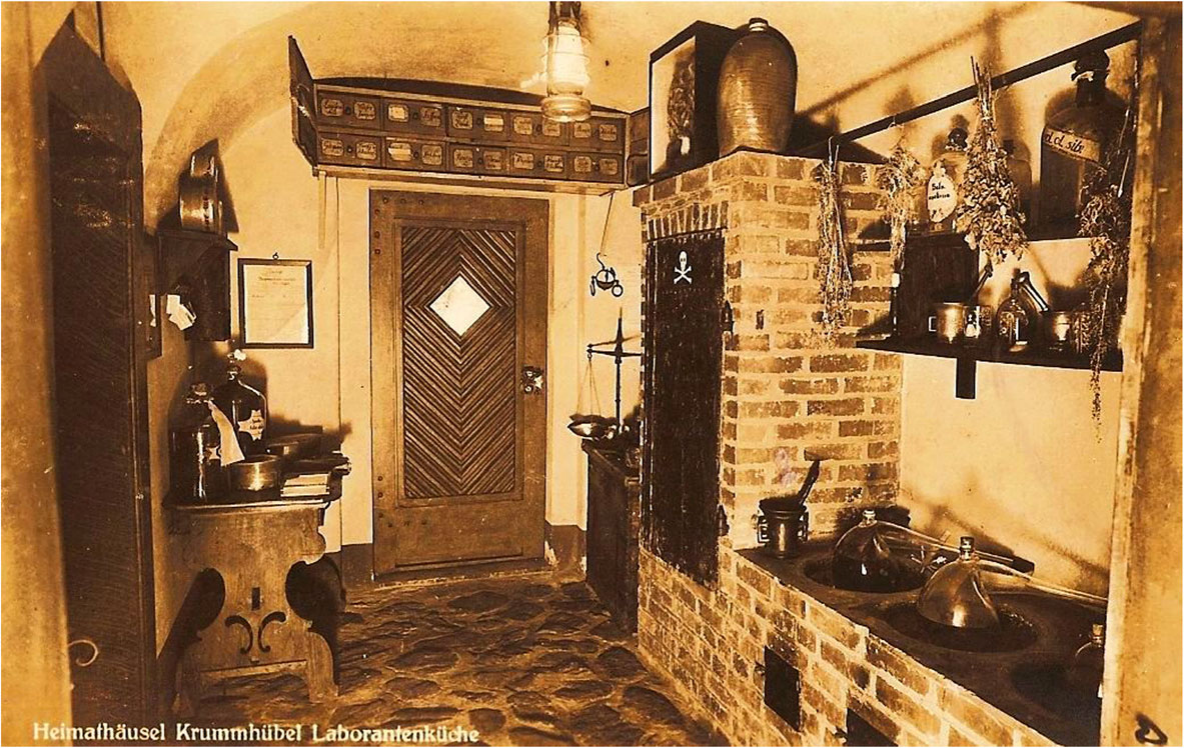
The museum dedicated to the herbalists from Krummhübel. Postcard from 1938 (collection of K. Spałek)

The beginnings of the production of herbal medicinal mixtures in the Sudety Mountains are related by multiple sources. One of them speaks of two protestants, well-known medics from Prague, who fled to the town of Krummhübel in the Sudety Mountains in the late seventeenth century to avoid punishment for participation in a bloody duel. Niclaus and Solomon found refuge in the house of Melchior Grossmann, where they established the first pharmacy in Krummhübel and introduced their saviour and his friend Jonas Exner to the art of producing herbal ointments, powders and tinctures [19–24]. At the end of the seventeenth century, Krummhübel had 57 households, of which about 40 were inhabited by herbalists, also known as “Laboranten” (in German). For this reason, Krummhübel was called “the village of pharmacists” [23–30]. The herbalists’ houses were log cabins with pitched roofs, timber framing and very distinctive interiors. The largest room on the ground floor, built of granite stones, was a laboratory with a large kitchen oven and distillation equipment (Fig. 1).

In the adjacent room, the medicinal raw material was produced and portioned. A side chamber, where cabinets, barrels, crates and shelves were located, served as a warehouse for storing products, and an airy loft served as a drying room for herbs. The dried products were stored in small free-standing structures due to the danger of fire [23, 31]. Next to the laboratory building, there was a backyard with medicinal plants, where, among others, the following plants were grown: *Plantago lanceolata* L., *Pimpinella anisum* L., *Menyanthes trifoliata* L., *Artemisia absinthium* L., *Centaurium erythraea* Rafn. subsp. *erythraea*, *Verbascum densiflorum* Bertol., *Carum carvi* L., *Trigonella foenum-graecum* L., *Valeriana officinalis* L., *Achillea millefolium* L., *Linum usitatissimum* L., *Alcea rosea* L. var. *nigra* Cav., *Taraxacum* spp., *Calendula officinalis* L., *Althaea officinalis* L., *Polygonum aviculare* L., *Rosa canina* L., *Ruta graveolens* L., *Salvia officinalis* L. and *Thymus pulegioides* L. Shrubs were also popular: *Viburnum opulus* L. and *Sambucus nigra* L. The most widespread plant, used for the production of many medicines, was *Digitalis purpurea* L., which still commonly grows on the slopes of the Sudety Mountains [23, 25, 32–34]. The other most popular plants used to manufacture medicaments were *Carlina acaulis* L.*, Primula elatior* (L.) Hill, *Arnica montana* L., *Lilium martagon* L., *Rhodiola rosea* L. and *Crocus* sp., i.e. probably *C. sativus* L. There are no voucher specimens associated with this study, so precautions in the identification of plant taxa in old written documents discussed by Łuczaj [35] were taken. Herbalists from Krummhübel enriched many drugs with powder roots of *Mandragora officinarum* L. [23], a plant species from the Solanaceae family, originating from the Mediterranean area and the Middle East and also grown in Krummhübel [36]. The following raw materials were used in therapeutics of the Mediterranean countries: the root *Mandragorae radix* and the herb *Mandragorae herba*, both of which contain tropane alkaloids. The root has anaesthetic and hypnotic properties, while the herb, with a lower content of alkaloids, is used in homoeopathy as a drug of analgesic properties for rheumatic diseases. *Mandragora officinarum* is one of the plants with the longest history of use in phytotherapy. It has already been mentioned in Egyptian papyri from 3000 years BC [4, 37, 38]. In the Middle Ages, a great cult developed around this plant as a panacea for any disease and a magical agent. At the end of the sixteenth century, the species disappeared from most of the drug stores and were obtained from the official European herbal market [36]. However, herbalists from Krummhübel used it until the end of the seventeenth century [25]. They were also the first in central Europe to use the herb *Drosera rotundifolia* L.—*Droserae herba* as a medicinal agent that had already been confirmed by Rittman [25]. Earlier, alchemists had examined the secretions of this plant’s glandular hairs that digest insects as they searched for preparations that could produce gold or a youth elixir. It also belonged to the group of so-called sacred herbs. Nowadays, this species is strictly protected by law in Poland, and the material for medicinal use comes from import only. Extracts of *Droserae herba* include derivatives of naphthoquinone, flavonoids and organic acids, which have antibacterial and antispastic properties [6, 7, 39]. The herbalists from Krummhübel also introduced *Rhodiola rosea* to phytotherapy in the Sudety region [25]. In medical practice, the rhizome of this species is now used, *Rhizoma Rhodiolae*, which has stimulating properties and enhances concentration and increases physical activity. It is used to treat tiredness, neurosis and anaemia [5, 39]. For these disorders, it was used in the form of mixtures by the Krummhübel herbalists [25].

To describe medicines, the herbalists used notes, armorials and prescriptions. Latin names were used to protect their trade secrets. Knowledge of Latin was required during the masters exam for herbalists, which was introduced around the year 1700. It was then that herbalists from Krummhübel and the surrounding area formed the common herbalists’ guild, one of the first in Central Europe. Medicinal knowledge was usually kept in secret by family members and passed from one generation to another. In the eighteenth century, Krummhübel herbalists manufactured more than 200 proprietary medicines [22–25, 40]. We confirmed that they were the first in the Sudetes who applied medicinal mixtures to the treatment of diseases (using, among others, plants; oils; powdered minerals, including rock crystal and amethyst; and animal formulations—adder venom, deer horns, toads, salamanders and frogs), and they left their medical legacy in writing [23] in contrast to previous herbalists, who only indicated the use of individual plant species for specific diseases. (The first researcher who published data on the distribution of medicinal plants and their therapeutic properties from the described area was the renaissance physicist Caspar Schwenckfeld from Hirschberg. The naturalist, however, did not provide and did not use any medicinal mixtures [1].) Medications from Krummhübel were mixed with water, wine, honey, oils, salts, acids and alcohol distilled in local or domestic distilleries [31, 37]. For instance, the tincture of *Arnica montana* was used as a painkiller and anti-inflammatory drug against digestive system disorders as well as used externally for bruises, frostbite and open wounds [23].

However, not all of these plants can be found in the recipes that have survived to this day (e.g. *Arnica montana*, *Rhodiola rosea* or *Mandragora officinarum*) [23]. Nevertheless, it was confirmed that specimens of these species so far are stored within the collections of the Museum of Sports and Tourism in Karpacz, and they certainly were used by the herbalists from Krummhübel.

The reign of the House of Hapsburg in Hirschberg brought no restrictions on the herbalists’ activities. However, the situation changed after 1740, when the area came under the rule of Prussia. As the popularity of the goods produced by the herbalists of Krummhübel increased, resentment and jealousy of doctors and pharmacists rose as well, since they considered them charlatan family clans who made fortunes from human illnesses [23, 31]. The desire to limit their activities also resulted from the Prussian administration’s wish to organise, e.g. health care, on a national level. The office of the Collegium Medicum et Sanitatis (Royal Council of Physicians and Pharmacists) intervened to limit the activity of the herbalists, and the production of herbal medicines by so-called laboratory workers was then only possible with an official license, and not based on the rules of the guild. The Act of 1740, issued by the Prussian government, reduced the number of legally operating herbalists to 30 persons only. To obtain a license, one had to wait for the death of a guild member and go through a long official procedure. In 1796, the herbalists’ guild comprised 27 members [23, 24]. The leaders of the guild were Christian Ignatius Exner (guild master), Benjamin Gottlieb Exner (guild chief) and Johannes Christoph Grossmann (guild chief assistant). The oldest known book by Krummhübel herbalists was produced in 1792 [22–25] and contained 150 recipes for mixtures made from local medicinal plants. From the late eighteenth century onwards, the herbalists encountered more and more difficulties. In 1796, the Prussian government allowed them to produce and sell only 46 medicines [23, 24]. Junker [24] provides a full list of them: (1) *Aqua apoplectica alba s. pauperum*, *weißes Schlagwasser*; (2) *Aqua apoplectica rubra*, *rotes Schlagwasser*; (3) *Balsamus anglicus*, *englischer Haupt- und Universalbalsam*; (4) *Balsamus embryonum liquidus*, *stärkender Kinderbalsam*; (5) *Balsamus sulpburis*; (6) *Balsamus vitae*, *Lebensbalsam*; (7) *Elixir pectorale*; (8) *Elixir proprietatis Paracelsi*; (9) *Elixir vitrioli Mynsichti*; (10) *Elixir uterinum*; (11) *Essentia absynthii composita*; (12) *Essentia alexipharmaca*; (13) *Essentia amara*; (14) *Essentia antidysenterica*, *Ruhrtropfen*; (15) *Essentia carminativa*; (16) *Essentia castorei*; (17) *Essentia corticum aurantiorum*; (18) *Essentia dulcis*; (19) *Essentia lignorum*; (20) *Essentia myrrhae*; (21) *Essentia rhei amara*; (22) *Essentia stomacbica composita*, *stärkende Gall- und Magentropfen*; (23) *Essentia succini*; (24) *Essentia absynthii simplex*; (25) *Liquor anodynus mineralis Hofmanni*; (26) *Mixtura symplex*; (27) *Morsuli anthelmintici*; (28) *Pulvis anthelminticus*; (29) *Pulvis bezoardicus*; (30) *Pulvis dentifriticus*, *Zahnpulver*; (31) *Pulvis marchionis*, *Marggrafenpulver*; (32) *Pulvis sternutatorius viridis*, *Hauptpulver*; (33) *Pulvis vitae*; (34) *Species zum Brust- und Blutreinigungsthee*; (35) *Spiritus comu cervi*; (36) *Spiritus matricalis*; (37) *Spiritus melissae compositus*; (38) *Spiritus nitri dulcis*; (39) *Spiritus salis ammoniaci aromatica*; (40) *Spiritus salis ammoniaci volatilis*; (41) *Spiritus tartari*; (42) *Spiritus theriacalis*; (43) *Tinctura bezoardica*; (44) *Tinctura coralliorum*; (45) *Tinctura laxans*; and (46) *Tinctura antimonii tartarisata.*

All 46 known recipes were provided by Reitzig [23], because he was studying the original manuscripts and recipes of the herbalists from Krummhübel, located in the then pre-war museum [41]. These manuscripts have not survived—they were lost in the war turmoil, which was confirmed directly by the Museum of Sports and Tourism in Karpacz and by the local libraries.

In 1797, licensed pharmacists persuaded the Prussian government to withdraw the privilege of selling the so-called drop of Krummhübel at fairs; it was one of the best-known medicines produced by the herbalists [31, 37]. In 1799, information was provided about a complementary treatment in the Warmbrunn spa by an anonymous relation of the practitioner. A herbalist with the initials P.I. was described, and during his presence at the spa, he was offering medicinal herbal mixtures to patients on request [23].

Despite the growing administrative difficulties, the popularity of medications from Krummhübel continued to increase. Among others, the eminent writer and representative of German Romanticism, Johann Wolfgang von Goethe, took an interest in medicinal herbs [42, 43]. Riesengebirge (in Polish Karkonosze, in the Western Sudetes) potions were sold in Austria, the Czech Republic, Poland and Russia, and at the turn of the nineteenth century, some of them could also be purchased in Scandinavia and England. In 1810, in the Sudetes, a company named W. Koerner & Co. was founded, which specialised in the production of liqueurs and tinctures prepared from Sudetic herbs [23, 24]. In subsequent years, the pharmacist herbalists of Krummhübel were affected by further restrictions. They were suspected of practicing black magic, secret cults, alchemy and possessing devil’s knowledge. In 1809, the authorities of Legnica Province (in German Kreis Liegnitz) banned the herbalists from conducting door-to-door trade. In the period from 1831 to 1832, a cholera epidemic raged in Central Europe, also reaching the Sudetes [31]. Fears of the disease were so great that the government decided to reach out for help from the Krummhübel herbalists. Carl Traugott Ende, who came from a family with a long tradition of herbalists, prepared medications for patients and was a member of the anti-cholera epidemic committee [23]. Despite this, the administrative restrictions on herbalists were restored after the end of the plague [42, 43]. In 1843, a royal edict was issued to limit the allowable number of simple Riesengebirge (Karkonosze) herbal medicines from 46 to 21. The list of 21 medicinal preparations approved in 1845 by the district doctor Dr. Schaeffer is as follows [23]: (1) *Aqua apoplectica alba s. pauperum*; (2) *Aqua apoplectica rubra (Schlagwasser)*; (3) *Balsamum anglicus*, *englischer Haupt- und Universalbalsam*; (4) *Balsamum vitae*, *Lebensbalsam*; (5) *Elixir pektorale*, *brustelixir*; (6) *Essentia amara*; (7) *Essentia carminativa*; (8) *Essentia corticum aurantiorum*; (9) *Essentia dulcis*; (10) *Essentia lignorum*; (11) *Essentia rhei Amara*, *bittere Rhabarber Tinktur*; (12) *Essentia stomacbica composita*, *stärkende Gallund Magentropfen*; (13) *Liquor anodynus, mineralis Hoffmanni Hoffmannsche Tropfen*; (14) *Pulvis sternutatorius viridis*, *Hauptpulver*; (15) *Pulvis Vita*, *Lebenspulver*; (16) *Species pectorales*, *Brust- und Blutreinigungsthee*; (17) *Spiritus melissae compositus*, *Karmelitenwasser*; (18) *Spiritus salis ammoniaci*, *aromaticus sive Spirit. volatilis oleosus Sylvii*; (19) *Spiritus salis ammoniaci volatilis*; (20) *Spiritus nitri dulcis*; and (21) *Tinctura Coralliorum*, *Corallen Tinktur.*

For the preparation of medicaments, only 24 strictly specified types of fruits and barks, 20 types of roots, 16 species of herbs (= aerial parts), 10 seeds and flowers and 2 species of timber were allowed [23]. In 1843, the Prussian government stopped issuing new licenses for herbal practices, which was the beginning of the end of the herbalists’ activity. In the Riesengebirge (Karkonosze), the herbalists’ art began to fade away in the second half of the nineteenth century. It was still possible to find some isolated cases of treatment using local herbs in later years, albeit only on a small scale. The last herbalist of the herbalists’ guild died on 28 March 1884 [23, 44].

The heritage of the herbalists from Krummhübel, regarding the use of medicinal plants and their mixtures, remained, mainly in the Sudety Mountains, until the beginning of the twentieth century, especially in folk medicine. *Drosera rotundifolia* may be presented as an example. It was used in the form of infusions for poor digestion, whooping cough and sclerosis by the residents of the Masyw Ślęży Mountains (in German Zobten-Gebirge) until the beginning of the twentieth century, although the species has not been found in the area so far [45].

The good reputation of Krummhübel herbalists and their gardens with medicinal plants is evidenced by the fact that they were visited by famous German botanists, including Max von Uechtritz [46].

Research on the activities of herbalists of Krummhübel was conducted by Will Erich Peuckert (1895–1969), a world-famous German ethnographer and ethnologist. In 1934, Peuckert became a professor at the Universität Breslau (University of Wrocław) and created a museum dedicated to the Krummhübel herbalists [23], which was, however, closed in the 1950s. Nowadays, an exhibition on pharmacist workers, including numerous exhibits, is held by the Museum of Sports and Tourism in Karpacz, which is the successor of the pre-war museum [47, 48].

## Material and methods

Based on 46 recipes by Krummhübel herbalists, re-written by Reitzig [23], we performed the analysis of use reports of drugs, which included plant taxa and other constituents such as animal formulations, fungi, inorganic and organic substances, minerals and tinctures (with alcohol/spirit) and elixirs (without alcohol/spirit). For each usage mentioned in the text, we recorded (i) the putative botanical identity of the taxon; (ii) the plant family or origin of other than the plant constituent; (iii) the reported plant part; (iv) the number of the recipe; (v) the name of the recipe; (vi) the vernacular name of the ingredient; (vii) the described symptom, ailment or specific use; (viii) our modern (viz. biomedical) interpretation of the described symptom or ailment; (ix) the mode of administration; and (x) the category of use under which we filed the specific use. Each recorded combination of the variables was counted as one individual (therapeutic) use report.

The following 10 plant parts or products were differentiated: barks, exudates (incl. gums, resins and saps), flowers (incl. inflorescences and parts thereof), fruits (incl. parts thereof), herbs (= aerial parts, incl. branches and shoots), leaves, oils (e.g. linseed oil), seeds, subterranean parts (incl. bulbs, rhizomes, roots and tubers) and wood. If there was no information on which plant part was used, it was qualified as an herb. The modes of administration were divided into two groups: internal (e.g. drops, many tinctures) and external (e.g. ointments and poultices). Use reports were classified into organ-, symptom- and ailment-defined categories of use, largely following the bioprospecting-oriented classification scheme proposed by Staub et al. [10]. The applied 15 categories of use citations comprise the following: andrology (incl. male fertility and venereal diseases: gonorrhoea, syphilis), antidotes (internally applied), cardiovascular problems, dermatology (e.g. tumours, injuries and wounds), fever, gastroenterology (e.g. appetite, intestinal obstruction, lithiasis liver and tympanites), gynaecology (incl. female fertility and venereal diseases: gonorrhoea, syphilis), musculoskeletal ailments (e.g. cramps, gout, rheumatism, scurvy and spasms), neurology (incl. psychosomatic ailments), oral cavity (e.g. dentistry and stomatitis), others (“internal wounds” and “for breast” but others than connected with respiratory system), parasites (e.g. anthelmintics), respiratory system (e.g. cleansing lungs and the upper respiratory tract, plague, tuberculosis) and urology (e.g. diuretics, lithiasis and kidneys).

This work also aimed at recalling the activities of Krummhübel herbalists and their input into the medical use reports of plants at that time. Taking this into account, we present selected species of medicinal plants and mixtures used by them, based on their recorded recipes. We selected the plant species that were most often used in mixtures and which were simultaneously growing in the medicinal plant gardens of Krummhübel herbalists. To achieve all these goals, we translated available source texts, including books, articles from magazines and guides as well as press notes on the activity of Krummhübel herbalists, from German.

We compared the medicinal plants of Krummhübel herbalists with the medicinal plant lists of Europe by cross-checking the species used in manuscripts and regional surveys, including Matthioli [11], Schwenckfeld [3], Mattuschka [12], Kneipp [13], Fischer (1930s) [14] and Madaus [15]. Matthioli’s book [11] is one of the most popular ethnobotanical studies and describes medicinal species; it was translated into a few languages. Schwenckfeld’s publication [3] constitutes the fullest analysis of therapeutic properties of the warm springs in Warmbrunn (Cieplice) as well as the plants used in spa and healing treatments [49]. Mattuschka is known for the work on the natural history of plants native to Silesia, in which he indicates species with medicinal properties [12]. Kneipp, one of the founders of the naturopathic medicine movement, developed his “Nature Cure” form of therapy based on subalpine plants from the Allgäu in Bavaria, which were used in folk medicine, and based on those, he cultivated in the garden [50, 51]. Fischer’s data (1930s) comprise the most complete information on folk botany, with nearly 250 plant species, used in the folk culture in the whole area of pre-World War II, Poland, which also includes the present Western Ukraine and parts of Belarus and Lithuania [14]. In his book, a German medical doctor, Madaus [15], discusses homoeopathic products and their use as therapeutic agents.

The paper follows the newest guidance referring to the analysis of historical texts [52]. Plant identifications were established by cross-checking the names and descriptions in the *Flora Europaea* [53] with the confirmed data that the individual species were growing in the area at that time [32, 33] or/and were cultivated in Krummhübel herbalists gardens [46] or/and are stored within the collections of the Museum of Sports and Tourism in Karpacz; thus, they certainly were used by the Krummhübel herbalists. Species names were checked against The Plant List 1.1 [54], and family names follow the Angiosperm Phylogeny Group IV [55].

## Results

### Patterns of the diversity of drugs

In total, among 46 drugs, 70 plant taxa were recorded. Of these, 52 taxa, included in 29 drugs, could be identified to the species and 18 taxa in 13 drugs were identified to the genus level. For 3 ingredients of plant origin, which were included in 5 drugs, no taxa identification was possible. Besides herbal, other constituents were used, including animal formulations (e.g. castoreum, corals, deer antlers, earthworms, scorpions, snakes), fungi, inorganic and organic substances (e.g. calcium, Sp[iritus] Vitrioli, Sp. Nitri dulcis, Sp. Nitri, Sp. Tartari, wax) and minerals (e.g. potash, pumice, salt) as well as elixirs and tinctures. These components were exclusively used in 13 drugs.

**Fig. 2:**
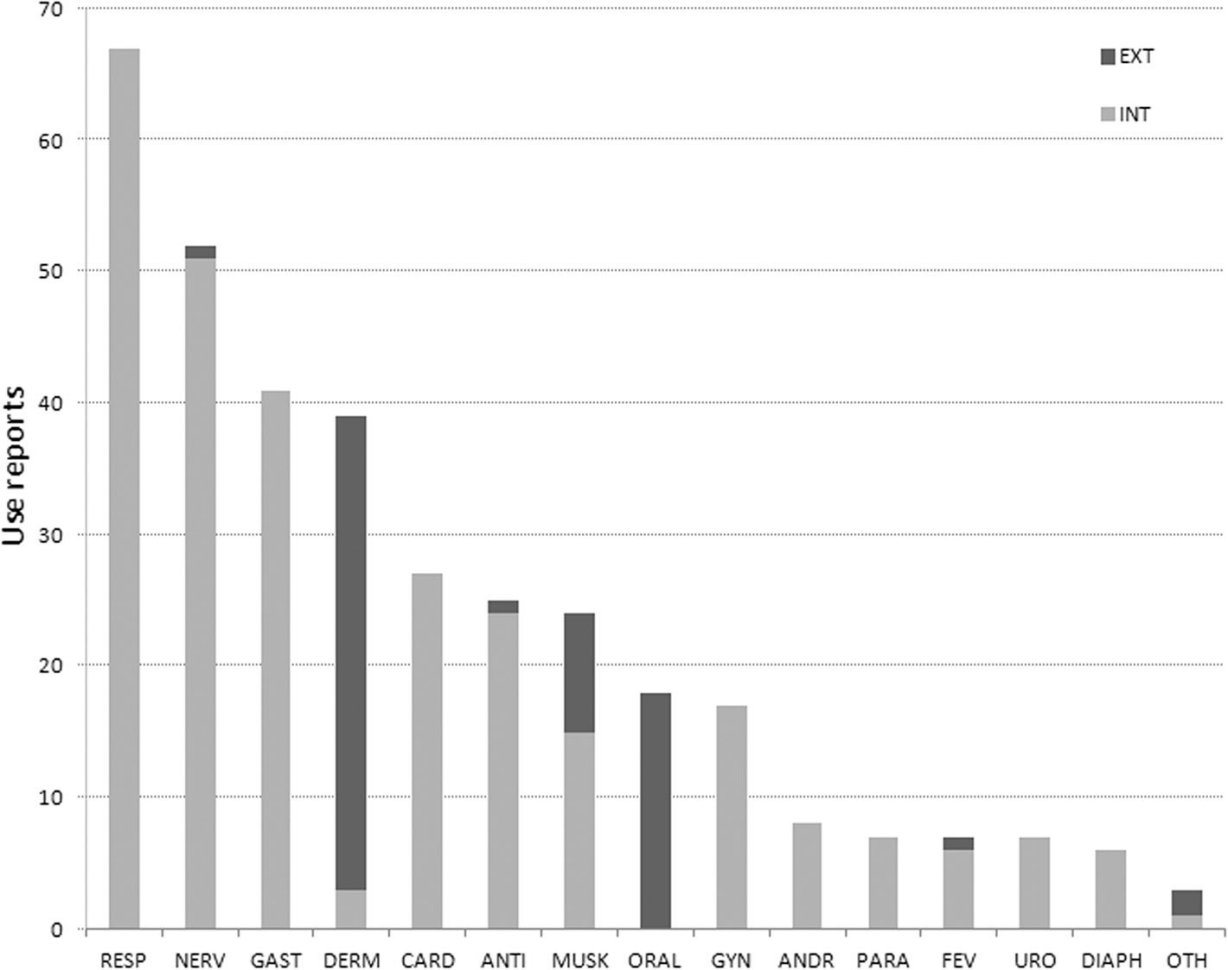
Number of use reports by category of use and mode of administration (*N* = 348). ANDR, andrology; ANTI, antidotes; CARD, cardiovascular problems; DERM, dermatology; DIAPH, diaphoretic; FEV, fever; GAST, gastroenterology; GYN, gynaecology; MUSK, musculoskeletal ailments; NERV, neurology; ORAL, oral cavity; OTH, others; PARA, parasites; RESP, respiratory system; URO, urology

Overall, 348 use reports (i.e. unique combinations of a specific taxon or another origin of a constituent, plant part, route of administration and specific use in individual recipes) were recorded. Internal applications, mainly as drops in tea or water (283 use reports), prevail over external applications such as ointments or poultices (69 use reports) (Fig. 2). Seventy-four records have no reported uses, i.e. when Reitzig’s [23] original did not clearly state the ailment they were used. The same remark applies to the lacking mode of administration (61 records). A full dataset of the recorded plant taxa, plant parts and other constituents used, as well as the therapeutic uses, is presented in Additional file 1: Table S2 and at the end of this paper.

**Fig. 3:**
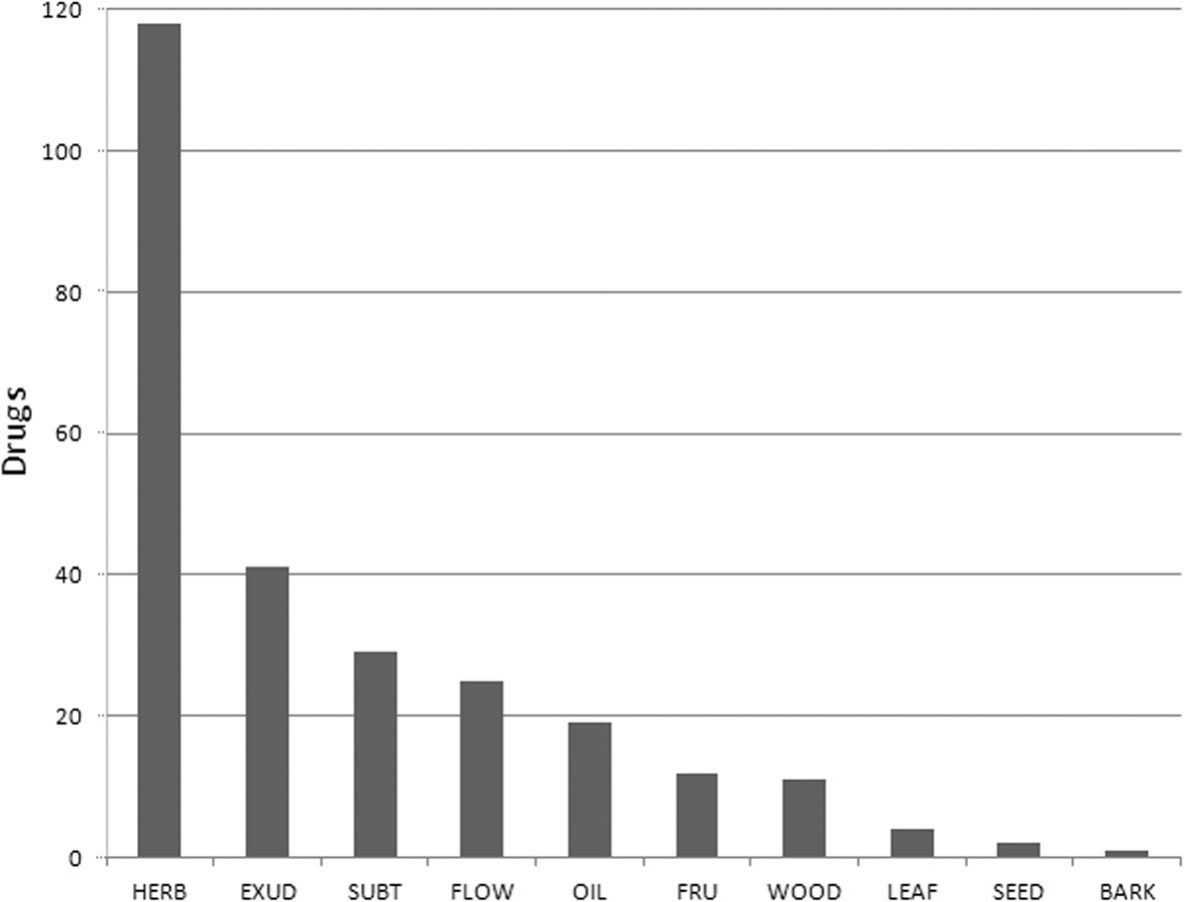
Number of drugs by plant part (*N* = 262). BARK, barks; EXUD, exudates; FLOW, flowers; FRU, fruits; HERB, herbs (= aerial parts); LEAF, leaves; OIL, oils; SEED, seeds; SUBT, subterranean parts; WOOD, wood

More than 45% of drugs stemmed from herbs (= aerial parts) (118), while the remaining percentage consisted of exudates (41), subterranean organs (29), flowers (25) and other less frequent plant parts (Fig. 3). The drugs were derived from members of 32 vascular plant families, with Asteraceae (9 taxa; 22 drugs), Fabaceae (8 taxa; 33 drugs) and Apiaceae (7 taxa; 26 drugs) being the most frequent ones.

**Fig. 4:**
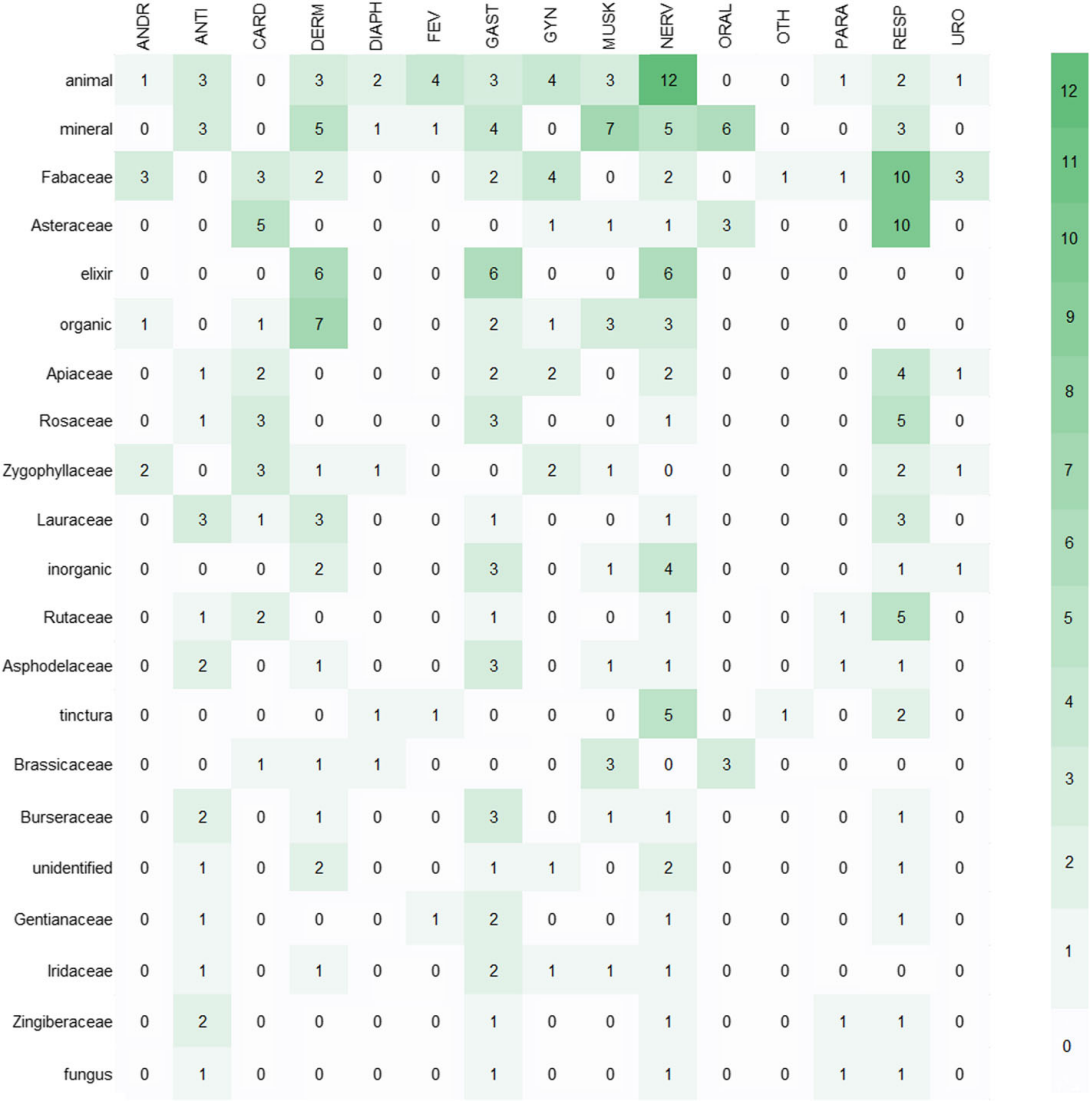
Quantification of use reports by category of use and the most important botanical families or other constituent origins. The categories of use are abbreviated following the legend of Fig. 2

**Fig. 5:**
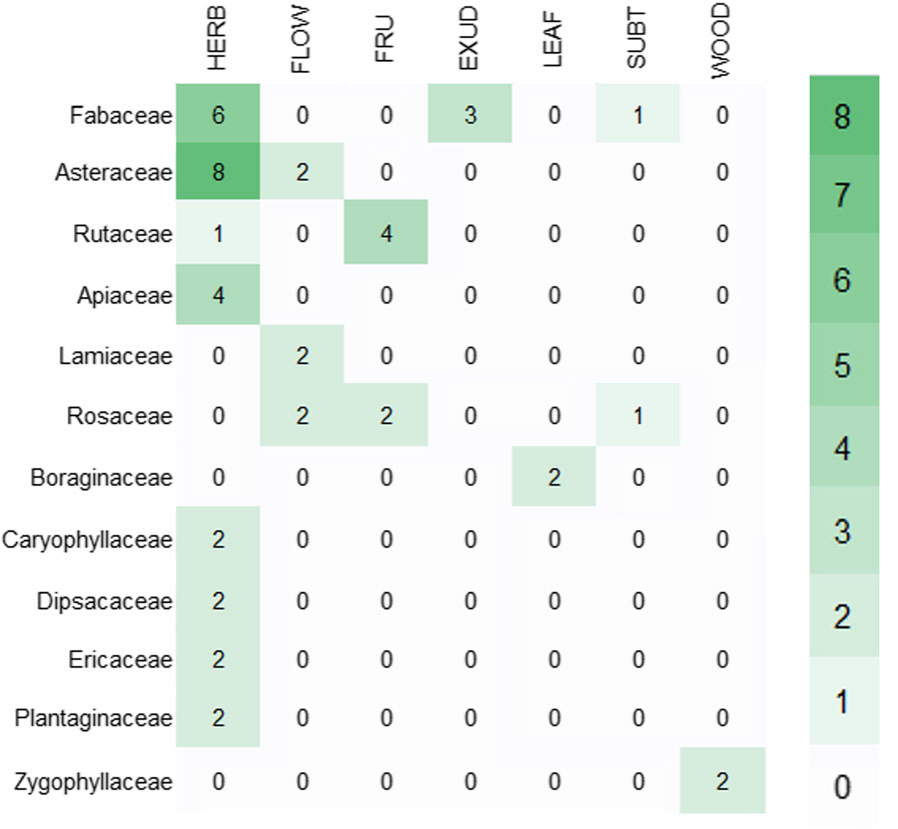
Quantification of use reports in the categories of respiratory system by plant part and botanical family. Plant part abbreviations follow the legend of Fig. 3

To characterise the therapeutic preferences, the associations between taxonomy, plant part or other constituent origins and categories of use were analysed (Figs. 4 and 5). The most frequently cited constituents were those obtained from animals and minerals. Animal formulations were recommended for neurology (12), gynaecology (4) and fever (4), while minerals were suggested for musculoskeletal ailments (7), oral cavity (6), neurology (5) and dermatology (5). Among the plant families, Fabaceae species were relatively frequently cited for respiratory system (10) and gynaecology (4), as well as Asteraceae for respiratory system (10) and cardiovascular problems (5).

The use citations for respiratory system issues are the most frequent ones (67; Fig. 2) and cover ailments concerning the lungs (21) and the upper respiratory tract (21) as well as plague (17). The remedies for these conditions are taxonomically diverse, including 7 different drugs derived from 34 plant taxa. Herbs of the families Asteraceae and Fabaceae account for a comparably high number of use reports in this category (8 and 6, respectively; Fig. 5) and include *Achillea* spp., *Cyanus segetum* Hill. (= *Centaurea cyanus* L.), *Matricaria* spp., *Glycyrrhiza glabra* L., *Hedysarum* spp. and *Ononis spinosa* L.

The category of nervous system and psychosomatic disorders (52; Fig. 2) includes plague (17), stroke (9), pain of the body (5), headache (3), brain disease (3) or postpartum discomfort (3). The most frequently used constituents were those not obtained from plants, but from animals (12), or were elixirs (6) and minerals (5).

Gastrointestinal use reports (41; Fig. 2) mainly comprise stomach and intestine problems (29) but also refer to appetite (9). Apart from the prevalence of elixirs (6) and minerals (4) in drugs, some use citations are based on Asphodelaceae (3), Burseraceae (3) and Rosaceae (3) species such as *Aloë* spp., *Commiphora* spp., *Potentilla erecta* (L.) Räusch. and *Rosa* spp.

Dermatology is the fourth largest category of use reports (39; Fig. 2) and mainly covers injuries and wounds (19) as well as applications for cleaning the head (5) and drying out feet (5). Organic substances (7) and elixirs (6) were the most frequently mentioned drugs. Among the plant families, Lauraceae oils (3) and Oleaceae exudates (3), including species such as *Cinnamomum camphora* (L.) J. Presl and *Olea europaea* L., were mostly recommended for uses.

The drugs mentioned for cardiovascular problems (6) are mainly remedies for blood purification (3), while 2 are recommended against oedema (i.e. wood of *Guaiacum officinale* L. and Sp. Tartari (= Weinstein-Geist)) and flowers of *Rosa* spp. as a heart tonic.

### Sample recipes and plant species

**Fig. 6:**
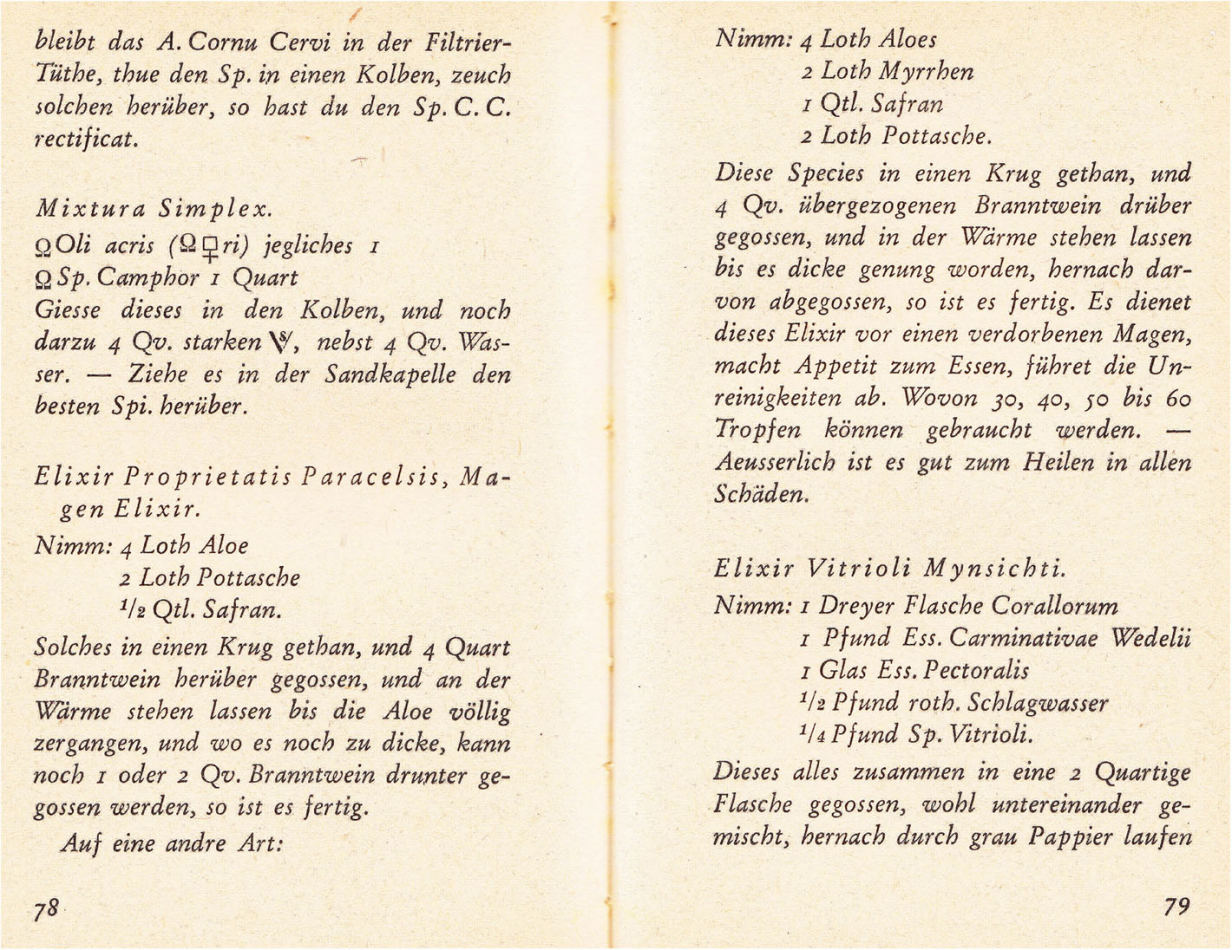
Original recipe using saffron (bottom part of page 78 and top part of page 79, from Reitzig [23]). Abbreviations: 1 Loth—about one large spoon (in Prussia, this equalled 14.606 g); 1 Quintl.—1/4 of Loth, which was about 3.651 g; Qv.—250 ml. [Elixir Proprietatis Paracelsis, stomach elixir—take 4 spoons of aloe (*Aloë vera* (L.) Burm.f.), 2 spoons of potash, 1/8 spoon of saffron (*Crocus* probably *sativus*). Put it in a pitcher and pour 4 quarts [4 × 1.14504 l] of strong spirit, then leave it in a warm place until aloe will completely dissolve, or, if it is still visible, you can add 250 ml or 500 ml of strong spirit until it is ready [i.e. well dissolved]. In a different way: take 4 spoons of aloe, 2 spoons of myrrh, 1/4 spoon of saffron and 2 spoons of potash. Put these species in a pitcher and add 1 l of strong spirit; leave it in a warm place until it is thick enough and pour off when ready. This elixir is served against stomach problems, increases appetite and detoxifies the body. You can take it starting from 30, 40, 50 up to 60 drops. Externally, it is good for healing any injuries.]

The extract of *Gentiana lutea* L.*—Essentia Gentianae* was recommended in the absence of appetite and for stomach pains. This plant was confirmed to be cultivated by the Krummhübel herbalists in their pharmaceutical gardens [46]. The original recipe was as follows: “Nimm 2 Loth gröblich gestossene Enzian-Wurzel, 1 Qv. starken Branntwein, halte es in der Wärme bis es sich gefärbet, hernach seige sie durch grau Pappier.*—Dienet in Schwachheit des Magens*, *bey allen 3 und 4 tägigten Fiebern 30 bis 40 Tropfen gebraucht”* ([23]; explanation of abbreviations in the caption of Fig. 6) [Take 2 spoons of coarsely minced [great yellow] gentian root (*Gentiana lutea*), 250 ml of strong spirit, and keep it in a warm place until it is dyed; afterwards, seep it through grey paper—serve it in weakness of the stomach, using 30 to 40 drops on all 3 and 4 days of fever.]

An extract from *Crocus* (probably) *sativus* was used by Riesengebirge (Karkonosze) herbalists as a spice and a dye (saffron). The extract also helped against digestive system diseases and circulatory problems. When dosed appropriately, it was considered an aphrodisiac and even a hallucination-inducing agent. Some quantitative recipes for this plant are also preserved (Fig. 6, from Reitzig [23]).

**Fig. 7:**
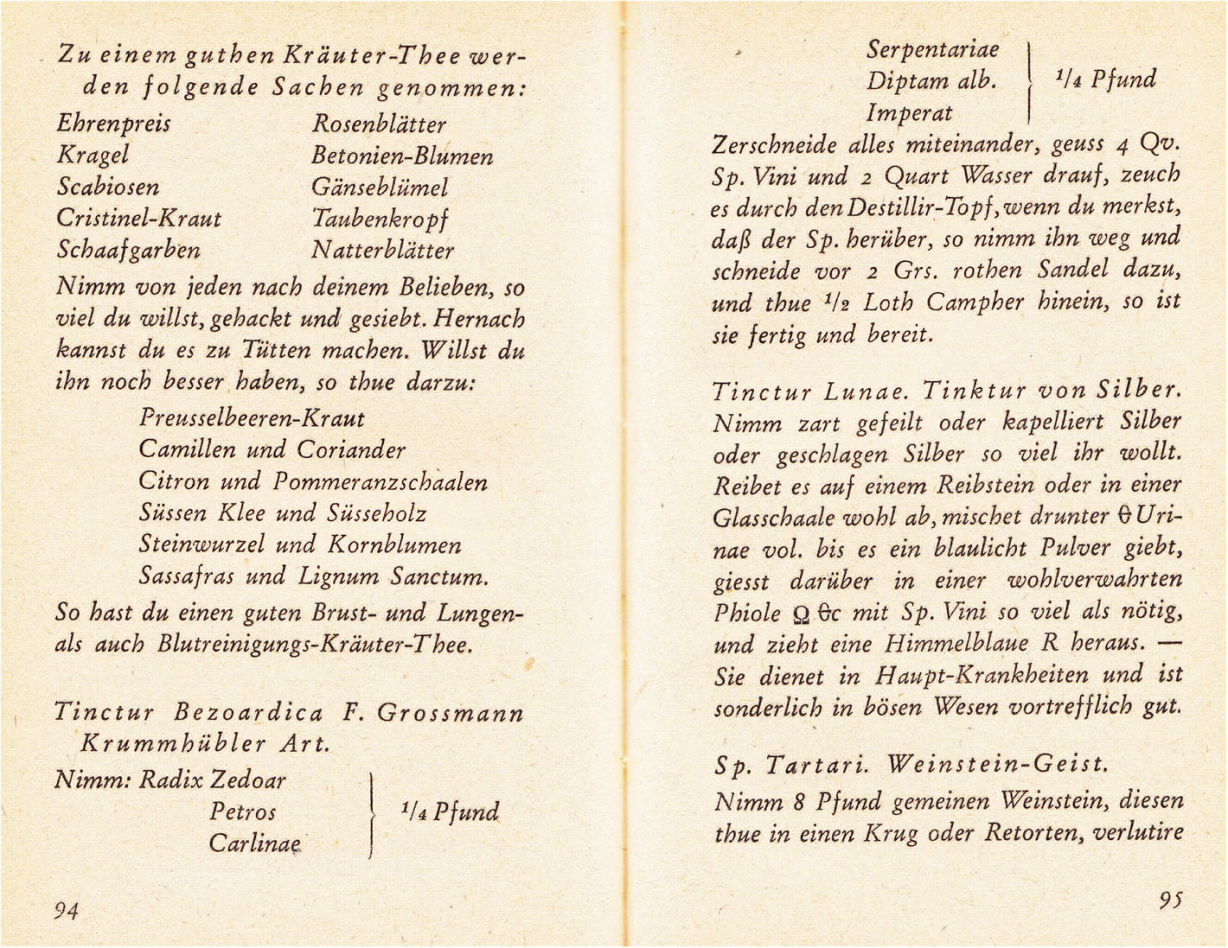
Original recipe using *Carlina acaulis* (bottom part of page 94 and top part of page 95, from Reitzig [23]). Abbreviations: 1 Loth—about 1 large spoon (in Prussia, this equalled 14.606 g); Qv.—250 ml; Grs.—gram [g]. [Tinctur Bezoardica of F. Grossmann in the way of Krummhübel—take Zedoary root (white turmeric, Zedoariae radix, *Curcuma zedoaria* (Christm.) Roscoe), parsley root (Petros [elini] radix, *Petroselinum crispum* (Mill.) Fuss), stemless carline thistle root (*Carlinae radix*, *Carlina acaulis*)—all together 125 g, as well as black cohosh root (*Serpentariae radix*, *Actaea racemosa* L. = *Cimicifuga racemosa* (L.) Nutt.), burning bush root (*Dictamni albus radix*, *Dictamnus albus* L.), masterwort root (Imperat [oriae] radix, *Peucedanum ostruthium* (L.) W.D.J. Koch = *Imperatoria ostruthium* L.)—all together 125 g. Chop everything together, add 1 l of Spiritus vini and 2.273 l of water, then pass it through the distiller glass. When the spirit has evaporated, the distillation can be finished and 2 g of sliced red sandal wood with half a spoon of camphor can be added. It is ready to use.]

A tincture made from the root of *Carlina acaulis* (a common plant in the area), recommended for digestion, was also appreciated for its antibacterial and antipyretic properties (Fig. 7). Sulphuric acid, manufactured in the Sudetes until the early nineteenth century, was of great importance to the production of herbal potions [22–25]. In the valley of the Kamienna river, there was a facility that produced sulphuric acid from pyrite shale. The term “vitriol” was the essence of the alchemical formulas, contained in the motto: *visita interiorem terrae rectificando invenies operae lapidem* (i.e. descend into the belly of the Earth, and in distilling you will find the stone of the work) [38, 56].

## Discussion

By using a systematic data extraction technique, we elucidated several salient therapeutic patterns in preserved recipes of Krummhübel herbalists. These include the importance of Fabaceae plants for respiratory system diseases and gynaecology, as well as Asteraceae for respiratory system and cardiovascular problems. Generally, the use citations for respiratory system issues are the most frequent ones and cover ailments concerning the lungs and the upper respiratory tract as well as plague. The remedies for these conditions are taxonomically diverse, including 7 different drugs derived from 34 plant taxa. Gastrointestinal use reports mainly comprise stomach and intestine problems but also refer to appetite and are based on Asphodelaceae, Burseraceae and Rosaceae species. Moreover, animal formulations were recommended for neurology, gynaecology and fever, while minerals were suggested for musculoskeletal ailments, oral cavity, neurology and dermatology.

This confirms that medicinal plants were commonly used in ethnomedicine for centuries, because they were the only drugs available to residents of many regions. Knowledge about their healing properties was passed down orally from generation to generation [57]. The past importance of mountain species and the frequent uses against envenomations and intoxications mirror the closer interaction of past societies with their biological environment and different ecological, epidemiological and hygienic conditions.

In the fifteenth century, the first herbaria and also herbals, written by doctors or other professionals, began to appear in Europe [57]. These items, however, do not take into account knowledge about medicinal plant mixtures provided by folk therapists. The phenomenon of herbalists from Krummhübel is connected with the fact that these unprofessional therapists left their knowledge on plant mixtures in a written form to be used for centuries in traditional folk medicine in the Sudety region.

The history of herbalists from Krummhübel shows that historical events, in this case, the disappearance of a well-established knowledge, has a lot to teach us now, such as regulations and restrictions kill traditions, which can also happen nowadays. An example may be the Kneipp phytotherapy method, recognised and currently used in health resort treatments. This method was known in the folk medicine of the Allgäu region in Southern Germany, and in the nineteenth century, it was not allowed as a therapy for a relatively long time (e.g. [58–61]).

The systematic analysis of herbal texts offers unique insights into past herbal medicine [10]. Thus, we also confirm the suggestion of Staub et al. [10] that those drugs with discontinued use might represent interesting starting points for drug discovery and the evaluation of old herbal medicine, especially that the information on this subject was strictly protected and included in the professional secrecy of the Krummhübel herbalists’ guild, not available to outsiders for centuries.

### Medicinal plants of Krummhübel herbalists used in other ethnobotanical studies

The highest share of the flora documented as medicinal plants by Krummhübel herbalists was recorded in Madaus [15] and Matthioli [11] and constituted 76.4 and 66.7%, respectively. This indicates that many plants used in medical treatment by Krummhübel herbalists were also known in other regions and in different periods. The medicinal plants, documented in all publications considered, included *Angelica* spp., *Carlina acaulis* L., *Gentiana* spp., *Juniperus* spp., *Rosa* spp. and *Veronica* spp. (Table 1). All of them are native or indigenous plants occurring in Central Europe. Additionally, *Gentiana* spp., *Juniperus* spp. and *Rosa* spp. were used for medicinal purposes through all other time periods in Switzerland [62]. While Polish inhabitants of the Carpathians, among others, valued *Angelica archangelica* L. and *Carlina acaulis* L. [57]. In the contemporary literature, only the use of *Veronica officinalis* is mentioned, but the use of *Veronica chamaedrys* and *V. beccabunga* is documented in ethnobotanical studies [62], and different *Veronica* species were cultivated in local Silesian gardens [46].

**Table 1:** Medicinal plants listed in Krummhübel herbalist recipes (seventeenth to nineteenth centuries) and their occurrences in manuscripts and regional surveys, including Matthioli [11], Schwenckfeld [3], Mattuschka [12], Kneipp [13], Fischer [14] and Madaus [15]

Krummhübel herbalists, seventeenth to nineteenth centuries	Matthioli (1563) [11]	Schwenckfeld (1607) [3]	Mattuschka (1779) [12]^a^	Kneipp (1892) [13]	Fischer (1930's) [14]^b^	Madaus (1938) [15]
*Achillea* spp.	+ (as Ptarmica)	−	+ (as *A. millefolium*, *A. ptarmica*)	+	+ (mainly as *A. millefolium* L., rare *A. ptarmica* L.)	+ (as A. ptarmica, A. millefolium*, A. moschata)
*Actaea racemosa* L. = *Cimicifuga racemosa* Nutt.	−	−	−	−	+ (as *A. spicata* L.)	+
*Aloë* spp.	+	+ (as Aloes Balsam)	−	+	+ (as *A. succotrina* Lam.)	+ (many species*)
*Anacyclus pyrethrum* (L.) Lag. = *Anacyclus officinarum* Hayne	−	−	−	−	−	+*
*Angelica* spp.	+ (as Angelica maior, A. minor)	+ (as *A. erratica*)	+ (as *A. syluestris*)	+	+ (as *A. archangelica* L., *A. sylvestris* L.)	+ (many species*)
*Artemisia vulgaris* L.	+ (as Artemisia)	−	+	+	+	+*
*Bellis perennis* L.	+ (as Bellis minor)	+ (as *B. minima*)	+	−	+	+*
*Carlina acaulis* L.	+ (as Chamaeleon albus) 261	+	+	+	+	+
*Cinnamomum camphora* (L.) J.Presl	+ (as camphora)	−	−	−	+	+*
*Cinnamomum verum* J.Presl	−	−	−	−	+	+ (as C. zeylanicum*)
*Citrus* × *aurantium* L.	+	−	−	−	−	+*
*Citrus limon* (L.) Osbeck	+	−	−	−	−	+ (as *C. limonum*)
*Citrus* spp.	+ (as Citria malus)	−	−	−	−	+ (many other species)
*Cochlearia officinalis* L.	−	−	+	+	−	+*
*Commiphora* spp.	−	−	−	−	−	+ (as *C. abyssinica*, *C. mukul, C. myrrha*)
*Copaifera officinalis* L.	−	−	−	−	−	−
*Coriandrum sativum* L.	+ (as Coriandrum)	−	−	−	−	+
*Crocus* (probably) *sativus* L.	+ (as Crocus florens)	−	−	−	+	−
*Curcuma zedoaria* (Christm.) Roscoe	+	−	−	−	−	+*
*Cyanus segetum* Hill. = *Centaurea cyanus* L.	+ (as Cyanus minor)	−	+	−	−	+*
*Dictamnus albus* L.	+	−	−	−	−	+
*Dorstenia contrajerva* L.	−	−	−	−	+	−
*Drimys winteri* J.R. Forst. & G. Forst.	−	−	−	−	−	−
*Echium vulgare* L.	+ (as Echium)	−	−	−	−	−
*Elettaria cardamomum* (L.) Maton	+ (as C. minus, C. medium, C. maius)	−	−	−	−	+
*Ferula assa-foetida* L.	+ (as Ferula = Nathex)	−	−	−	−	+*
*Foeniculum vulgare* Mill.	+ (as Foeniculum)	−	−	+	+	+*
*Gentiana* spp.	+ (as Gentiana minor = cruciata, Gentiana (probably lutea))	+ (as *G. major cœruleo flore*, *G. minor punctato flore*)	+ (as *G. lutea, G. centaurium*, *G. amarella*)	+	+	+ (many species*)
*Glycyrrhiza glabra* L.	+ (as Glycyrrhiza liquiritia)	−	−	−	−	+*
*Guaiacum officinale* L.	+ (as Lignum guaiacum)	−	−	−	−	+*
*Guaiacum sanctum* L. or *G. officinale* L.	+ (as Lignum guaiacum)	−	−	−	−	−
*Hedysarum* spp.	−	−	−	−	−	−
*Helleborus niger* L.	+	−	+	−	−	+*
*Indigofera* spp.	−	−	−	−	−	+
*Inula helenium* L.	+ (as Elenium)	−	+	−	+	+*
*Juniperus* spp.	+ (as Juniperus, as Sabina)	+	+ (as *I. communis*)	+	+ (as *J. communis* L.)	+ (many species*)
*Laurus nobilis* L.	+ (as Laurus)	+ (as *L. Alexandrina* Matthioli)	−	+	−	+*
*Lavandula* spp.	+	−	−	+	−	+*
*Linum usitatissimum* L.	+ (as Linum)	−	+	+	+	+*
*Matricaria* spp.	+ (as Camomilla)	−	+ (as *M. chamomilla*, *M. parthenium*)	+	+ (as *M. chamomilla* L.)	+ (as M. chamomilla*, M. discoidea*)
*Melissa officinalis* L.	+ (as Melissa)	−	+ (as *M. calamintha*)	+	+	+*
*Mentha aquatica* L. var. *crispa* (L.) Benth.	+ (as Mentha aquatica)	−	−	+	*+* (as *Mentha* spp.)	+
*Myristica fragrans* Houtt.	−	−	−	−	−	−
*Myroxylon balsamum* (L.) Harms	−	−	−	−	−	+*
*Nasturtium officinale* R.Br.	+ (as Nasturcium aquaticum)	−	−	+	+	+*
*Olea europaea* L.	+ (as Olea domestica)	−	−	+	−	−
*Ononis spinosa* L.	+ (as Ononis)	−	+ (as *O. aruensis*)	−	+	+*
*Origanum majorana* L.	+ (as Maiorana)	−	−	−	−	+*
*Persicaria bistorta* (L.) Samp. = *Polygonum bistorta* L.	+ (as Bistorta)	−	+	−	+	−
*Petasites* sp.	+ (as Petasites falso dieta)	+	+ (as *Tussilago petasites*)	−	−	+ (many species*)
*Petroselinum crispum* (Mill.) Fuss.	+ (as Pertoselinum vulgare)	−	−	−	−	+ (as P. sativum*)
*Peucedanum ostruthium* (L.) W.D.J. Koch = *Imperatoria ostruthium* L.	−	−	+	−	−	+
*Pimpinella anisum* L.	+ (as Anisum)	+ (as *P. major* L. Huds, *P. minorcrispa*)	−	+	+	+*
*Piper longum* L.	−	−	−	−	−	−
*Potentilla erecta* (L.) Räusch.	+ (as Tormentilla)	−	+ (as *Tormentilla erecta*)	−	−	+ (as P. tormentilla*)
*Pterocarpus santalinus* L. fil.	−	−	−	+	−	−
*Pulicaria vulgaris* Gaertn.	−	−	−	−	+	−
*Pyrus* sp.	+	−	−	−	+ (as *P. communis* L.)	+ (as P. malus*)
*Rheum rhabarbarum* L.	+	−	−	−	−	−
*Rosa* spp.	+	+ (as *R. alpina*, *R. rubra*)	+ (as *R. canina*, *R. alba*)	+	+	+ (many species*)
*Rosmarinus officinalis* L.	+ (as Rosmarinus coronaria)	+ (as *R. sylvaticus*)	−	+	+	+*
*Sassafras* spp.	−	−	−	−	−	+*
*Scabiosa* spp.	+ (as Scabiosa minor)	−	+ (as *Scabiosa succisa* and *S. aruensis*)	−	+ (as *Succisa pratensis* Moench = *Scabiosa succisa* L.)	+ (different species*)
*Senna* spp.	+ (as Sena)	−	−	−	−	+*
*Silene baccifera* (L.) Roth = *Cucubalus baccifer* L.	−	−	−	−	−	−
*Stachys officinalis* (L.) Trevis = *Betonica officinalis* L.	+ (as Betonica)	−	+	−	+	+
*Styrax* spp.	+	−	−	−	−	−
*Syzygium aromaticum* (L.) Merr. & L.M.Perry	−	−	−	−	−	−
*Vaccinium vitis*-*idaea* L.	−	−	+	+	+ (as *V. myrtillus* L.)	+*
*Veronica* spp.	+ (as Veronica mas, V. foemina)	+	+ (as *V. officinalis, V. beccabunga*)	+	+	+ (many species*)
*Viola* spp.	+ (as Viola purpurea)	−	+ (as *V. odorata*)	+	+	+ (many species*)
*Zingiber officinale* Roscoe	+ (as Zinziber)	−	−	−	+	+*
Σ = 72	Σ = 48	Σ = 11	Σ = 25	Σ = 24	Σ = 33	Σ = 55

*Denotes to Madaus [15], asterisk shows the description of use or recipe; no asterisk—plant was only listed

^a^The register of Mattuschka [12] includes only species listed by the author as having medicinal properties

^b^Based on Kujawska et al. [14]

On the other hand, in therapeutic mixtures of Krummhübel herbalists, eight taxa were exclusive, including mainly exotic plants such as *Copaifera officinalis* L., *Drimys winteri* J.R. Forst. & G. Forst., *Hedysarum* spp., *Myristica fragrans* Houtt., *Piper longum* L., *Silene baccifera* (L.) Roth and *Syzygium aromaticum* (L.) Merr. & L.M. Perry. Although they originate from various parts of the world, they were quite frequently used in several remedies by Krummhübel herbalists and are still highly important herbs in modern folk medicine. The oleoresin of *Copaifera* trees has been widely used in Neotropical regions for thousands of years and remains a popular treatment for a variety of ailments [63]. One of the most ancient and valuable spices of the Orient is clove (*Syzygium aromaticum* (L.) Merr. & L.M. Perry), which has a wide spectrum of biological activity [64]. The exotic plants used by Krummhübel herbalists may also refer to the scholarly origin of their knowledge. This, and the considerable overlap of the nomenclature with the old herbals, indicates that the recipes might have been originated (even if later modified) from the monastic tradition dating back to at least the sixteenth century [65, 66], and they may go back even to antiquity. For example, by producing and marketing drugs to the public, Italian Renaissance nuns both augmented the medical resources available in urban society and acquired roles of public significance beyond the spiritual realm [66](Table 2).

**Table 2:** Compact list of the vascular plant uses described in “Die Laboranten von Krummhübel” [23]

Plant taxon	Family or origin	Part	No. of reci–pe	Name of recipe	Vernacular	Ailment	Ailment_interpretation	Mode	Category
–	Animal	–	1	Sp. Lumbricorum, Regenwurm-Spiritus	Regenwürmer (Gemeine Regenwurm)	–	–	–	–
–	Tinctura	–	1	Sp. Lumbricorum, Regenwurm-Spiritus	Sp. Vini	–	–	–	–
–	Animal	–	2	Sp. Cornu Cervi, Hirschhorn-Geist	Hirschhorn	–	–	–	–
–	Tinctura	–	3	Mixtura siplex	Oli acris	–	–	–	–
*Cinnamomum camphora* (L.) J.Presl	Lauraceae	OIL	3	Mixtura siplex	Sp. Camphor	–	–	–	–
–	Mineral	–	4	Elixir Proprietatis Paracelsis, Magen Elixir	Pottasche (potash)	verdorbenen Magen	Against stomach problems	INT	GAST
–	Mineral	–	4	Elixir Proprietatis Paracelsis, Magen Elixir	Pottasche	führet die Unreinigkeiten ab	Detoxifies the body	INT	ANTI
–	Mineral	–	4	Elixir Proprietatis Paracelsis, Magen Elixir	Pottasche	zem Heilen in ellen Schäden	Good for healing of any injuries	EXT	DERM
–	Mineral	–	4	Elixir Proprietatis Paracelsis, Magen Elixir	Pottasche (potash)	macht Appetit zum Essen	Increases appetite	INT	GAST
*Aloë* spp.	Asphodelaceae	HERB	4	Elixir Proprietatis Paracelsis, Magen Elixir	Aloe	verdorbenen Magen	Against stomach problems	INT	GAST
*Aloë* spp.	Asphodelaceae	HERB	4	Elixir Proprietatis Paracelsis, Magen Elixir	Aloe	führet die Unreinigkeiten ab	Detoxifies the body	INT	ANTI
*Aloë* spp.	Asphodelaceae	HERB	4	Elixir Proprietatis Paracelsis, Magen Elixir	Aloe	zem Heilen in ellen Schäden	good for healing of any injuries	EXT	DERM
*Aloë* spp.	Asphodelaceae	HERB	4	Elixir Proprietatis Paracelsis, Magen Elixir	Aloe	macht Appetit zum Essen	Increases appetite	INT	GAST
*Commiphora* spp.	Burseraceae	EXUD	4	Elixir Proprietatis Paracelsis, Magen Elixir	Myrrhen	verdorbenen Magen	Against stomach problems	INT	GAST
*Commiphora* spp.	Burseraceae	EXUD	4	Elixir Proprietatis Paracelsis, Magen Elixir	Myrrhen	führet die Unreinigkeiten ab	Detoxifies the body	INT	ANTI
*Commiphora* spp.	Burseraceae	EXUD	4	Elixir Proprietatis Paracelsis, Magen Elixir	Myrrhen	zem Heilen in ellen Schäden	Good for healing of any injuries	EXT	DERM
*Commiphora* spp.	Burseraceae	EXUD	4	Elixir Proprietatis Paracelsis, Magen Elixir	Myrrhen	macht Appetit zum Essen	Increases appetite	INT	GAST
*Crocus* probably *sativus* L.	Iridaceae	FLOW	4	Elixir Proprietatis Paracelsis, Magen Elixir	Safran	verdorbenen Magen	Against stomach problems	INT	GAST
*Crocus* probably *sativus* L.	Iridaceae	FLOW	4	Elixir Proprietatis Paracelsis, Magen Elixir	Safran	führet die Unreinigkeiten ab	Detoxifies the body	INT	ANTI
*Crocus* probably *sativus* L.	Iridaceae	FLOW	4	Elixir Proprietatis Paracelsis, Magen Elixir	Safran	zem Heilen in ellen Schäden	Good for healing of any injuries	EXT	DERM
*Crocus* probably *sativus* L.	Iridaceae	FLOW	4	Elixir Proprietatis Paracelsis, Magen Elixir	Safran	macht Appetit zum Essen	Increases appetite	INT	GAST
–	Elixir	–	5	Elixir Vitrioli Mynsichti	Ess. Carminativae Wedelii	reiniget das Haupt	Cleans the head	EXT	DERM
–	Elixir	–	5	Elixir Vitrioli Mynsichti	Ess. Pectoralis	reiniget das Haupt	Cleans the head	EXT	DERM
–	Animal	–	5	Elixir Vitrioli Mynsichti	Corallorum	reiniget das Haupt	Cleans the head	EXT	DERM
–	Elixir	–	5	Elixir Vitrioli Mynsichti	roth. Schlagwasser	reiniget das Haupt	Cleans the head	EXT	DERM
–	Inorganic	–	5	Elixir Vitrioli Mynsichti	Sp. Vitrioli	reiniget das Haupt	Cleans the head	EXT	DERM
–	Animal	–	5	Elixir Vitrioli Mynsichti	Corallorum	trocknet die Füsse aus	Dries out the feet	EXT	DERM
–	Elixir	–	5	Elixir Vitrioli Mynsichti	Ess. Carminativae Wedelii	trocknet die Füsse aus	Dries out the feet	EXT	DERM
–	Elixir	–	5	Elixir Vitrioli Mynsichti	Ess. Pectoralis	trocknet die Füsse aus	Dries out the feet	EXT	DERM
–	Elixir	–	5	Elixir Vitrioli Mynsichti	roth. Schlagwasser	trocknet die Füsse aus	Dries out the feet	EXT	DERM
–	Inorganic	–	5	Elixir Vitrioli Mynsichti	Sp. Vitrioli	trocknet die Füsse aus	Dries out the feet	EXT	DERM
–	Animal	–	5	Elixir Vitrioli Mynsichti	Corallorum	macht Appetit	Increases appetite	INT	GAST
–	elixir	–	5	Elixir Vitrioli Mynsichti	Ess. Carminativae Wedelii	macht Appetit	increases appetite	INT	GAST
–	Elixir	–	5	Elixir Vitrioli Mynsichti	Ess. Pectoralis	macht Appetit	Increases appetite	INT	GAST
–	Elixir	–	5	Elixir Vitrioli Mynsichti	roth. Schlagwasser	macht Appetit	Increases appetite	INT	GAST
–	Inorganic	–	5	Elixir Vitrioli Mynsichti	Sp. Vitrioli	macht Appetit	Increases appetite	INT	GAST
–	Elixir	–	5	Elixir Vitrioli Mynsichti	Ess. Carminativae Wedelii	bewahret vor dem Schlage und der Schweren-Noth	Prevents stroke	INT	NERV
–	Elixir	–	5	Elixir Vitrioli Mynsichti	Ess. Pectoralis	bewahret vor dem Schlage und der Schweren–Noth	Prevents stroke	INT	NERV
–	Animal	–	5	Elixir Vitrioli Mynsichti	Corallorum	bewahret vor dem Schlage und der Schweren–Noth	Prevents stroke	INT	NERV
–	Elixir	–	5	Elixir Vitrioli Mynsichti	roth. Schlagwasser	bewahret vor dem Schlage und der Schweren–Noth	Prevents stroke	INT	NERV
–	Inorganic	–	5	Elixir Vitrioli Mynsichti	Sp. Vitrioli	bewahret vor dem Schlage und der Schweren–Noth	Prevents stroke	INT	NERV
–	Animal	–	5	Elixir Vitrioli Mynsichti	Corallorum	verwahret den ganzen Leib vol allen Schmerzen	Protects the whole body from all pain	INT	NERV
–	Elixir	–	5	Elixir Vitrioli Mynsichti	Ess. Carminativae Wedelii	verwahret den ganzen Leib vol allen Schmerzen	Protects the whole body from all pain	INT	NERV
–	Elixir	–	5	Elixir Vitrioli Mynsichti	Ess. Pectoralis	verwahret den ganzen Leib vol allen Schmerzen	Protects the whole body from all pain	INT	NERV
–	Elixir	–	5	Elixir Vitrioli Mynsichti	roth. Schlagwasser	verwahret den ganzen Leib vol allen Schmerzen	Protects the whole body from all pain	INT	NERV
–	inorganic	–	5	Elixir Vitrioli Mynsichti	Sp. Vitrioli	verwahret den ganzen Leib vol allen Schmerzen	Protects the whole body from all pain	INT	NERV
–	Animal	–	5	Elixir Vitrioli Mynsichti	Corallorum	stärket Magen und Eingeweide	Strengthens the stomach and intestines	INT	GAST
–	Elixir	–	5	Elixir Vitrioli Mynsichti	Ess. Carminativae Wedelii	stärket Magen und Eingeweide	Strengthens the stomach and intestines	INT	GAST
–	Elixir	–	5	Elixir Vitrioli Mynsichti	Ess. Pectoralis	stärket Magen und Eingeweide	Strengthens the stomach and intestines	INT	GAST
–	Elixir	–	5	Elixir Vitrioli Mynsichti	roth. Schlagwasser	stärket Magen und Eingeweide	Strengthens the stomach and intestines	INT	GAST
–	Inorganic	–	5	Elixir Vitrioli Mynsichti	Sp. Vitrioli	stärket Magen und Eingeweide	Strengthens the stomach and intestines	INT	GAST
–	Animal	–	6	Elixir Uterini Crolly	Castor od. Bibergeil	allen Mutterkrankenheiten	Postpartum discomfort	INT	NERV
–	Animal	–	6	Elixir Uterini Crolly	Castor od. Bibergeil	allen Mutterkrankenheiten	Postpartum discomfort	INT	GYN
?	?	EXUD	6	Elixir Uterini Crolly	Oleum Succini	allen Mutterkrankenheiten	Postpartum discomfort	INT	NERV
?	?	EXUD	6	Elixir Uterini Crolly	Oleum Succini	allen Mutterkrankenheiten	Postpartum discomfort	INT	GYN
*Artemisia vulgaris* L.	Asteraceae	HERB	6	Elixir Uterini Crolly	Artemisiae oder Beyfuss	allen Mutterkrankenheiten	Postpartum discomfort	INT	NERV
*Artemisia vulgaris* L.	Asteraceae	HERB	6	Elixir Uterini Crolly	Artemisiae oder Beyfuss	allen Mutterkrankenheiten	Postpartum discomfort	INT	GYN
*Crocus* probably *sativus* L.	Iridaceae	FLOW	6	Elixir Uterini Crolly	Safran	allen Mutterkrankenheiten	Postpartum discomfort	INT	GYN
*Crocus* probably *sativus* L.	Iridaceae	FLOW	6	Elixir Uterini Crolly	Safran	allen Mutterkrankenheiten	Postpartum discomfort	INT	NERV
*Pimpinella anisum* L.	Apiaceae	OIL	6	Elixir Uterini Crolly	Oleum anisi	allen Mutterkrankenheiten	Postpartum discomfort	INT	GYN
*Pimpinella anisum* L.	Apiaceae	OIL	6	Elixir Uterini Crolly	Oleum anisi	allen Mutterkrankenheiten	Postpartum discomfort	INT	NERV
*Pterocarpus santalinus* L. fil.	Fabaceae	HERB	6	Elixir Uterini Crolly	rothen Sandel	allen Mutterkrankenheiten	Postpartum discomfort	INT	GYN
*Pterocarpus santalinus* L. fil.	Fabaceae	HERB	6	Elixir Uterini Crolly	rothen Sandel	allen Mutterkrankenheiten	Postpartum discomfort	INT	NERV
–	Inorganic	–	7	Elixir anti Scorbutic: Elixir vor den Scharbock	Sp. Nitri dulcis	vor den Scharbock	Against scurvy	INT	MUSK
–	Mineral	–	7	Elixir anti Scorbutic: Elixir vor den Scharbock	Pottasche	vor den Scharbock	Against scurvy	INT	MUSK
Aloë spp.	Asphodelaceae	HERB	7	Elixir anti Scorbutic: Elixir vor den Scharbock	Aloe	vor den Scharbock	Against scurvy	INT	MUSK
*Cochlearia officinalis* L.	Brassicaceae	HERB	7	Elixir anti Scorbutic: Elixir vor den Scharbock	Sp. Cochlear	vor den Scharbock	Against scurvy	INT	MUSK
*Commiphora* spp.	Burseraceae	EXUD	7	Elixir anti Scorbutic: Elixir vor den Scharbock	Myrrhen	vor den Scharbock	Against scurvy	INT	MUSK
*Crocus* probably *sativus* L.	Iridaceae	FLOW	7	Elixir anti Scorbutic: Elixir vor den Scharbock	Safran	vor den Scharbock	Against scurvy	INT	MUSK
*Nasturtium officinale* R.Br.	Brassicaceae	HERB	8	Spirit. Cochlear: Löffel-Kraut-Geist	Brunnenkresse	Blutreinigung	Blood purification	INT	CARD
*Nasturtium officinale* R.Br.	Brassicaceae	HERB	8	Spirit. Cochlear: Löffel-Kraut-Geist	Brunnenkresse	treibt den Schweiss	Diaphoretic	INT	DIAPH
*Nasturtium officinale* R.Br.	Brassicaceae	HERB	8	Spirit. Cochlear: Löffel-Kraut-Geist	Brunnenkresse	widersteht der Fäule	Prevents ulcers	INT	DERM
*Nasturtium officinale* R.Br.	Brassicaceae	HERB	8	Spirit. Cochlear: Löffel-Kraut-Geist	Brunnenkresse	widersteht dem Scharbock	Prevents scurvy	INT	MUSK
*Guaiacum officinale* L.	Zygophyllaceae	WOOD	9	Sp. Sassafras. Franzosen-Holz-Geist	Franzosen-Holz	reiniget das Geblüth	Blood purification	INT	CARD
*Guaiacum officinale* L.	Zygophyllaceae	WOOD	9	Sp. Sassafras. Franzosen-Holz-Geist	Franzosen-Holz	treibt Schweiss	Diaphoretic	INT	DIAPH
*Guaiacum officinale* L.	Zygophyllaceae	WOOD	9	Sp. Sassafras. Franzosen-Holz-Geist	Franzosen-Holz	treibt Harn	Diuretic	INT	URO
*Guaiacum officinale* L.	Zygophyllaceae	WOOD	9	Sp. Sassafras. Franzosen-Holz-Geist	Franzosen-Holz	Wassersucht	Oedema	INT	CARD
*Guaiacum officinale* L.	Zygophyllaceae	WOOD	9	Sp. Sassafras. Franzosen-Holz-Geist	Franzosen-Holz	Gliederreissen	Rheumatism	INT	MUSK
*Guaiacum officinale* L.	Zygophyllaceae	WOOD	9	Sp. Sassafras. Franzosen-Holz-Geist	Franzosen-Holz	Krätze	Scabies	EXT	DERM
*Guaiacum officinale* L.	Zygophyllaceae	WOOD	9	Sp. Sassafras. Franzosen-Holz-Geist	Franzosen-Holz	Franzosen	Syphilis	INT	ANDR
*Guaiacum officinale* L.	Zygophyllaceae	WOOD	9	Sp. Sassafras. Franzosen-Holz-Geist	Franzosen-Holz	Franzosen	Syphilis	INT	GYN
–	Animal	–	10	Sp. Lumbricorum, Regenwürmer–Geist	Regenwürmer (Gemeine Regenwurm)	gegen Krampf	Against cramp (skurcz)	EXT	MUSK
–	Animal	–	10	Sp. Lumbricorum, Regenwürmer-Geist	Regenwürmer (Gemeine Regenwurm)	gegen Gebrechen der Nerven	Against nerve ailments	EXT	NERV
–	Animal	–	10	Sp. Lumbricorum, Regenwürmer–Geist	Regenwürmer (Gemeine Regenwurm)	gegen die Schlagflüss gerühmet	Against stroke	INT	NERV
–	Animal	–	10	Sp. Lumbricorum, Regenwürmer-Geist	Regenwürmer (Gemeine Regenwurm)	gegen die Schmerzlaufende Gicht	Against the painful gout	EXT	MUSK
–	Animal	–	10	Sp. Lumbricorum, Regenwürmer-Geist	Regenwürmer (Gemeine Regenwurm)	hat eine Schmerzstillende Kraft	Antiphlogistic	INT	FEV
–	Animal	–	10	Sp. Lumbricorum, Regenwürmer-Geist	Regenwürmer (Gemeine Regenwurm)	treibt Schweiss	Diaphoretic	INT	DIAPH
–	Animal	–	10	Sp. Lumbricorum, Regenwürmer-Geist	Regenwürmer (Gemeine Regenwurm)	treibt Harn	Diuretic	INT	URO
–	Animal	–	10	Sp. Lumbricorum, Regenwürmer-Geist	Regenwürmer (Gemeine Regenwurm)	Reissen in Gliedern	Rheumatism	EXT	MUSK
–	Animal	–	11	Sp. Viperarum, Natterngräten-Geist	Natterngräten	vor toller Hunde und giftiger Thiere Biss	Against bites of mad dogs and poisonous animals	INT	ANTI
–	Animal	–	11	Sp. Viperarum, Natterngräten-Geist	Natterngräten	vor toller Hunde und giftiger Thiere Biss	Against bites of mad dogs and poisonous animals	EXT	ANTI
–	Animal	–	11	Sp. Viperarum, Natterngräten-Geist	Natterngräten	hitzigen Fiebern	High fevers	INT	FEV
–	Animal	–	11	Sp. Viperarum, Natterngräten-Geist	Natterngräten	hitzigen Fiebern	High fevers	EXT	FEV
*Carlina acaulis* L.	Asteraceae	SUBT	12	Allgem. Bezoar. Tinct. - Nach Krummhübler Art.	Eberwurzel	–	–	–	–
*Cinnamomum camphora* (L.) J.Presl	Lauraceae	OIL	12	Allgem. Bezoar. Tinct. - Nach Krummhübler Art.	Campfer	–	–	–	–
*Dorstenia contrajerva* L.	Moraceae	SUBT	12	Allgem. Bezoar. Tinct. - Nach Krummhübler Art.	Ra. Bezoardica alba	–	–	–	–
*Petasites* sp.	Asteraceae	SUBT	12	Allgem. Bezoar. Tinct. - Nach Krummhübler Art.	Pestilenzwurzel	–	–	–	–
*Peucedanum ostruthium* (L.) W.D.J.Koch	Apiaceae	SUBT	12	Allgem. Bezoar. Tinct. - Nach Krummhübler Art.	Meisterwurzel	–	–	–	–
*Persicaria bistorta* (L.) Samp. = *Polygonum bistorta* L.	Polygonaceae	SUBT	12	Allgem. Bezoar. Tinct. - Nach Krummhübler Art.	Otterwurzel	–	–	–	–
–	Inorganic	–	13	Mixtura siplex	Sp. Nitri	–	–	–	–
–	Inorganic	–	13	Mixtura siplex	Sp. Tartari	–	–	–	–
–	Animal	–	14	Tinctura Castori, Bibergeil-Tinctur	Bibergeil	allen Mutterbeschwerungen	Postpartum discomfort	INT	NERV
–	Animal	–	14	Tinctura Castori, Bibergeil-Tinctur	Bibergeil	allen Mutterbeschwerungen	Postpartum discomfort	INT	GYN
*Pterocarpus santalinus* L. fil.	Fabaceae	HERB	14	Tinctura Castori, Bibergeil-Tinctur	rothen Sandel	allen Mutterbeschwerungen	Postpartum discomfort	INT	GYN
*Pterocarpus santalinus* L. fil.	Fabaceae	HERB	14	Tinctura Castori, Bibergeil–Tinctur	rothen Sandel	allen Mutterbeschwerungen	postpartum discomfort	INT	NERV
*Ferula assa–foetida* L.	Apiaceae	–	15	Ess. Asha foetida, Teufelsdreck–Essentz	Gummi asha foetida	–	–	EXT	–
*Ferula assa–foetida* L.	Apiaceae	EXUD	15	Ess. Asha foetida, Teufelsdreck-Essentz	Gummi asha foetida	dienet vor Milz	Spleen	INT	CARD
*Ferula assa–foetida* L.	Apiaceae	EXUD	15	Ess. Asha foetida, Teufelsdreck-Essentz	Gummi asha foetida	dienet vor Mutter	Uterus	INT	GYN
–	Animal	–	16	Ess. Castor, Bibergeil-Essentz	Bibergeil	stillet die Mutterbeschwerung und das böse Wesen	Calms down postpartum discomfort including depression	INT	NERV
–	Animal	–	16	Ess. Castor, Bibergeil-Essentz	Bibergeil	stillet die Mutterbeschwerung und das böse Wesen	Calms down postpartum discomfort including depression	INT	GYN
–	Animal	–	16	Ess. Castor, Bibergeil-Essentz	Bibergeil	curiret den Schlag	Heals stroke	INT	NERV
–	Inorganic	–	17	Schwarzenberger Gnad und Lebens-Balsam	Bals. Sulphur	viele Tugenden beygelegt werden	It has many advantages	–	–
?	Organic	–	17	Schwarzenberger Gnad und Lebens-Balsam	Oleum Petrae	viele Tugenden beygelegt werden	It has many advantages	–	–
?	?	EXUD	17	Schwarzenberger Gnad und Lebens-Balsam	Oleum Ther[eb]inth.	viele Tugenden beygelegt werden	It has many advantages	–	–
?	?	EXUD	17	Schwarzenberger Gnad und Lebens-Balsam	Ol. Succini	viele Tugenden beygelegt werden	It has many advantages	–	–
*Juniperus* spp.	Cupressaceae	EXUD	17	Schwarzenberger Gnad und Lebens-Balsam	Oleum Juniperi	viele Tugenden beygelegt werden	It has many advantages	–	–
?	?	EXUD	18	Engl. Balsam	Venetian Therebinth.	–	–	–	–
*Angelica* spp.	Apiaceae	HERB	18	Engl. Balsam	Angelica	–	–	–	–
*Cinnamomum verum* J.Presl	Lauraceae	BARK	18	Engl. Balsam	Zimmet (Zimt)	–	–	–	–
*Citrus limon* (L.) Osbeck	Rutaceae	FRU	18	Engl. Balsam	Citronschaal	–	–	–	–
*Citrus* spp.	Rutaceae	FRU	18	Engl. Balsam	Pomeranzenschaal	–	–	–	–
*Elettaria cardamomum* (L.) Maton	Zingiberaceae	HERB	18	Engl. Balsam	Cardemome	–	–	–	–
*Foeniculum vulgare* Mill.	Apiaceae	HERB	18	Engl. Balsam	Fenchel	–	–	–	–
*Indigofera* spp.	Fabaceae	HERB	18	Engl. Balsam	Balsam Indigo	–	–	–	–
*Inula helenium* L.	Asteraceae	HERB	18	Engl. Balsam	Alant	–	–	–	–
*Juniperus* spp.	Cupressaceae	HERB	18	Engl. Balsam	Wacholder	–	–	–	–
*Laurus nobilis* L.	Lauraceae	HERB	18	Engl. Balsam	Loorbeer	–	–	–	–
*Lavandula* spp.	Lamiaceae	HERB	18	Engl. Balsam	Lavendel	–	–	–	–
*Melissa officinalis* L.	Lamiaceae	HERB	18	Engl. Balsam	Melisse	–	–	–	–
*Mentha aquatica* L. var. *crispa* (L.) Benth.	Lamiaceae	HERB	18	Engl. Balsam	Krausemünze	–	–	–	–
*Myristica fragrans* Houtt.	Myristicaceae	FLOW	18	Engl. Balsam	Muscat-Blüthen	–	–	–	–
*Pimpinella anisum* L.	Apiaceae	HERB	18	Engl. Balsam	Anis	–	–	–	–
*Rosmarinus officinalis* L.	Lamiaceae	FLOW	18	Engl. Balsam	Rosmarin-Blüthen	–	–	–	–
*Syzygium aromaticum* (L.) Merr. & L.M.Perry	Myrtaceae	HERB	18	Engl. Balsam	Nelcken	–	–	–	–
–	Mineral	–	19	Ol. Phylosophorum seu Laterinum, Ziegel-Oel	neue Ziegel in Feuer recht glühend	erweichet und hat in harten Geschwulsten vortrefflichen Nutzen	Softens and has excellent benefits in hard tumours	EXT	DERM
*Linum usitatissimum* L.	Linaceae	OIL	19	Ol. Phylosophorum seu Laterinum, Ziegel-Oel	Oleum Lini od. Leinöl	erweichet und hat in harten Geschwulsten vortrefflichen Nutzen	Softens and has excellent benefits in hard tumours	EXT	DERM
*Copaifera officinalis* L.	Fabaceae	EXUD	20	Balsamus Copeive	Copeive	gegen den Tripper	Against gonorrhoea	INT	GYN
*Copaifera officinalis* L.	Fabaceae	EXUD	20	Balsamus Copeive	Copeive	gegen den Tripper	Against gonorrhoea	INT	ANDR
*Copaifera officinalis* L.	Fabaceae	EXUD	20	Balsamus Copeive	Copeive	gegen Saamenfluss	Against nocturnal emission	INT	ANDR
*Copaifera officinalis* L.	Fabaceae	EXUD	20	Balsamus Copeive	Copeive	gegen die Franzosen gerühmet	Aagainst syphilis	INT	ANDR
*Copaifera officinalis* L.	Fabaceae	EXUD	20	Balsamus Copeive	Copeive	gegen die Franzosen gerühmet	Aagainst syphilis	INT	GYN
*Copaifera officinalis* L.	Fabaceae	EXUD	20	Balsamus Copeive	Copeive	gegen brennenden Harn	Dysuria or painful urination	INT	URO
*Copaifera officinalis* L.	Fabaceae	EXUD	20	Balsamus Copeive	Copeive	in allen äusserlichen und innerlichen Verwundungen	In all external and internal wounds	EXT	DERM
*Copaifera officinalis* L.	Fabaceae	EXUD	20	Balsamus Copeive	Copeive	in allen äusserlichen und innerlichen Verwundungen	In all external and internal wounds	INT	OTH
*Copaifera officinalis* L.	Fabaceae	EXUD	20	Balsamus Copeive	Copeive	Steinschmerzen	Lithiasis	INT	GAST
*Copaifera officinalis* L.	Fabaceae	EXUD	20	Balsamus Copeive	Copeive	Steinschmerzen	Lithiasis	INT	URO
*Copaifera officinalis* L.	Fabaceae	EXUD	20	Balsamus Copeive	Copeive	Lungensucht	Tuberculosis	INT	RESP
*Myroxylon balsamum* (L.) Harms	Fabaceae	EXUD	21	Balsam Opo	–	vor langwieriges Keuchen	Against protracted wheezing	INT	RESP
*Myroxylon balsamum* (L.) Harms	Fabaceae	EXUD	21	Balsam Opo	–	heilet frische Wunden	Heals fresh wounds	EXT	DERM
*Myroxylon balsamum* (L.) Harms	Fabaceae	EXUD	21	Balsam Opo	–	dient zur Schwind- und Lungensucht	Tuberculosis	INT	RESP
*Citrus limon* (L.) Osbeck	Rutaceae	FRU	22	Kayserl. und Königl. Lebens-Pulver	Zitronenschaalen	–	–	–	–
*Drimys winter*i J.R. Forst. & G. Forst.	Winteraceae	HERB	22	Kayserl. und Königl. Lebens-Pulver	weiss Zimmet	–	–	–	–
*Foeniculum vulgare* Mill.	Apiaceae	HERB	22	Kayserl. und Königl. Lebens-Pulver	Fenchel	–	–	–	–
*Glycyrrhiza glabra* L.	Fabaceae	HERB	22	Kayserl. und Königl. Lebens-Pulver	Süssholz	–	–	–	–
*Inula helenium* L.	Asteraceae	HERB	22	Kayserl. und Königl. Lebens-Pulver	Alant	–	–	–	–
*Pimpinella anisum* L.	Apiaceae	HERB	22	Kayserl. und Königl. Lebens-Pulver	Anis	–	–	–	–
*Coriandrum sativum* L.	Apiaceae	HERB	23	Fein Schwarzenberger Haupt-Pulver	Coriander	–	–	–	–
*Foeniculum vulgare* Mill.	Apiaceae	HERB	23	Fein Schwarzenberger Haupt-Pulver	Fenchel	–	–	–	–
*Helleborus niger* L.	Ranunculaceae	HERB	23	Fein Schwarzenberger Haupt-Pulver	Niesewurzel	–	–	–	–
*Lavandula* spp.	Lamiaceae	HERB	23	Fein Schwarzenberger Haupt-Pulver	Lavendel	–	–	–	–
*Origanum majorana* L.	Lamiaceae	HERB	23	Fein Schwarzenberger Haupt-Pulver	Majoran	–	–	–	–
*Pimpinella anisum* L.	Apiaceae	HERB	23	Fein Schwarzenberger Haupt-Pulver	Anis	–	–	–	–
*Pulicaria vulgaris* Gaertn.	Asteraceae	HERB	23	Fein Schwarzenberger Haupt-Pulver	Cristinelkraut	–	–	–	–
*–*	Fungus	–	24	Wurm-Pulver	Lerchenschwamm	Wurm	Anthelmintic	INT	PARA
–	Animal	–	24	Wurm-Pulver	roth und weisse Korallen	Wurm	Anthelmintic	INT	PARA
Aloë spp.	Asphodelaceae	HERB	24	Wurm-Pulver	Aloes	Wurm	Anthelmintic	INT	PARA
*Curcuma zedoaria* (Christm.) Roscoe	Zingiberaceae	SEED	24	Wurm-Pulver	Zittwersaamen	Wurm	Anthelmintic	INT	PARA
*Dictamnus albus* L.	Rutaceae	HERB	24	Wurm-Pulver	weiss Diptam W.	Wurm	Aanthelmintic	INT	PARA
*Senna* spp.	Fabaceae	LEAF	24	Wurm-Pulver	Sennes-Blätter	Wurm	Anthelmintic	INT	PARA
*Viola* spp.	Violaceae	SUBT	24	Wurm-Pulver	Viol. Wurzel	Wurm	Anthelmintic	INT	PARA
–	Mineral	–	25	Zahn-Pulver	Bimstein	benimmt Scharbock	Against scurvy	EXT	MUSK
–	Mineral	–	25	Zahn-Pulver	gebrannt Alaun	benimmt Scharbock	Against scurvy	EXT	MUSK
–	Mineral	–	25	Zahn-Pulver	Bimstein	benimmt Mundfäule	Against stomatitis	EXT	ORAL
–	Mineral	–	25	Zahn-Pulver	gebrannt Alaun	benimmt Mundfäule	Against stomatitis	EXT	ORAL
–	Mineral	–	25	Zahn-Pulver	Bimstein	schwarze Zähne werden weiß	Black teeth turn white	EXT	ORAL
–	Mineral	–	25	Zahn-Pulver	gebrannt Alaun	schwarze Zähne werden weiß	Black teeth turn white	EXT	ORAL
–	Mineral	–	25	Zahn-Pulver	Bimstein	macht wackelnde Zähne feste	Makes wobbly teeth firm	EXT	ORAL
–	Mineral	–	25	Zahn-Pulver	gebrannt Alaun	macht wackelnde Zähne feste	Makes wobbly teeth firm	EXT	ORAL
*Anacyclus pyrethrum* (L.) Lag. = *Anacyclus officinarum* Hayne	Asteraceae	SUBT	25	Zahn-Pulver	Bertran Wurzel	benimmt Scharbock	Against scurvy	EXT	MUSK
*Anacyclus pyrethrum* (L.) Lag. = *Anacyclus officinarum* Hayne	Asteraceae	SUBT	25	Zahn-Pulver	Bertran Wurzel	benimmt Mundfäule	Against stomatitis	EXT	ORAL
*Anacyclus pyrethrum* (L.) Lag. = *Anacyclus officinarum* Hayne	Asteraceae	SUBT	25	Zahn-Pulver	Bertran Wurzel	schwarze Zähne werden weiß	Black teeth turn white	EXT	ORAL
*Anacyclus pyrethrum* (L.) Lag. = *Anacyclus officinarum* Hayne	Asteraceae	SUBT	25	Zahn-Pulver	Bertran Wurzel	macht wackelnde Zähne feste	Makes wobbly teeth firm	EXT	ORAL
*Myristica fragrans* Houtt.	Myristicaceae	FLOW	25	Zahn-Pulver	Muscat-Blüthen	benimmt Scharbock	Against scurvy	EXT	MUSK
*Myristica fragrans* Houtt.	Myristicaceae	FLOW	25	Zahn-Pulver	Muscat-Blüthen	benimmt Mundfäule	Against stomatitis	EXT	ORAL
*Myristica fragrans* Houtt.	Myristicaceae	FLOW	25	Zahn-Pulver	Muscat-Blüthen	schwarze Zähne werden weiß	Black teeth turn white	EXT	ORAL
*Myristica fragrans* Houtt.	Myristicaceae	FLOW	25	Zahn-Pulver	Muscat-Blüthen	macht wackelnde Zähne feste	Makes wobbly teeth firm	EXT	ORAL
*Nasturtium officinale* R.Br.	Brassicaceae	HERB	25	08	Brunnenkresse	benimmt Scharbock	Against scurvy	EXT	MUSK
*Nasturtium officinale* R.Br.	Brassicaceae	HERB	25	Zahn-Pulver	Brunnenkresse	benimmt Mundfäule	Against stomatitis	EXT	ORAL
*Nasturtium officinale* R.Br.	Brassicaceae	HERB	25	Zahn-Pulver	Brunnenkresse	schwarze Zähne werden weiß	Black teeth turn white	EXT	ORAL
*Nasturtium officinale* R.Br.	Brassicaceae	HERB	25	Zahn-Pulver	Brunnenkresse	macht wackelnde Zähne feste	Makes wobbly teeth firm	EXT	ORAL
*Syzygium aromaticum* (L.) Merr. & L.M.Perry	Myrtaceae	HERB	25	Zahn-Pulver	Nelcken	benimmt Scharbock	Against scurvy	EXT	MUSK
*Syzygium aromaticum* (L.) Merr. & L.M.Perry	Myrtaceae	HERB	25	Zahn-Pulver	Nelcken	benimmt Mundfäule	Against stomatitis	EXT	ORAL
*Syzygium aromaticum* (L.) Merr. & L.M.Perry	Myrtaceae	HERB	25	Zahn-Pulver	Nelcken	schwarze Zähne werden weiß	Black teeth turn white	EXT	ORAL
*Syzygium aromaticum* (L.) Merr. & L.M.Perry	Myrtaceae	HERB	25	Zahn-Pulver	Nelcken	macht wackelnde Zähne feste	Makes wobbly teeth firm	EXT	ORAL
–	Animal	–	26	Recept von einem besonderen Elixir	Biebergeil	In pest	Against plague	INT	NERV
–	Animal	–	26	Recept von einem besonderen Elixir	Biebergeil	In pest	Against plague	INT	RESP
*–*	Fungus	–	26	Recept von einem besonderen Elixir	Lerchenschwamm	In pest	Against plague	INT	NERV
–	Mineral	–	26	Recept von einem besonderen Elixir	Terra Sigill	In pest	Against plague	INT	NERV
–	Mineral	–	26	Recept von einem besonderen Elixir	Potaschen (potash)	In pest	Against plague	INT	NERV
*–*	Fungus	–	26	Recept von einem besonderen Elixir	Lerchenschwamm	In pest	Against plague	INT	RESP
–	Mineral	–	26	Recept von einem besonderen Elixir	Terra Sigill	In pest	Against plague	INT	RESP
–	Mineral	–	26	Recept von einem besonderen Elixir	Potaschen (potash)	In pest	Against plague	INT	RESP
–	Animal	–	26	Recept von einem besonderen Elixir	Biebergeil	herrl. Magen-Essenz	Against stomach problems	INT	GAST
*–*	Fungus	–	26	Recept von einem besonderen Elixir	Lerchenschwamm	herrl. Magen-Essenz	Against stomach problems	INT	GAST
–	Mineral	–	26	Recept von einem besonderen Elixir	Terra Sigill	herrl. Magen-Essenz	Against stomach problems	INT	GAST
–	Mineral	–	26	Recept von einem besonderen Elixir	Potaschen (potash)	herrl. Magen-Essenz	Against stomach problems	INT	GAST
–	Animal	–	26	Recept von einem besonderen Elixir	Biebergeil	Praeservativ vor alle Gifte	Protection against all poisons	INT	ANTI
*–*	Fungus	–	26	Recept von einem besonderen Elixir	Lerchenschwamm	Praeservativ vor alle Gifte	Protection against all poisons	INT	ANTI
–	Mineral	–	26	Recept von einem besonderen Elixir	Terra Sigill	Praeservativ vor alle Gifte	Protection against all poisons	INT	ANTI
–	Mineral	–	26	Recept von einem besonderen Elixir	Potaschen (potash)	Praeservativ vor alle Gifte	Protection against all poisons	INT	ANTI
?	?	?	26	Recept von einem besonderen Elixir	Theriac [as antidote to poisons, especially on viper venom]	In pest	Against plague	INT	NERV
?	?	?	26	Recept von einem besonderen Elixir	Theriac [as antidote to poisons, especially on viper venom]	In pest	Against plague	INT	RESP
?	?	?	26	Recept von einem besonderen Elixir	Theriac [as antidote to poisons, especially on viper venom]	herrl. Magen-Essenz	Against stomach problems	INT	GAST
?	?	?	26	Recept von einem besonderen Elixir	Theriac [as antidote to poisons, especially on viper venom]	Praeservativ vor alle Gifte	Protection against all poisons	INT	ANTI
Aloë spp.	Asphodelaceae	HERB	26	Recept von einem besonderen Elixir	Aloe	In pest	Against plague	INT	NERV
Aloë spp.	Asphodelaceae	HERB	26	Recept von einem besonderen Elixir	Aloe	In pest	Against plague	INT	RESP
Aloë spp.	Asphodelaceae	HERB	26	Recept von einem besonderen Elixir	Aloe	herrl. Magen-Essenz	Against stomach problems	INT	GAST
Aloë spp.	Asphodelaceae	HERB	26	Recept von einem besonderen Elixir	Aloe	Praeservativ vor alle Gifte	Protection against all poisons	INT	ANTI
*Angelica* spp.	Apiaceae	HERB	26	Recept von einem besonderen Elixir	Angelica	In pest	Against plague	INT	NERV
*Angelica* spp.	Apiaceae	HERB	26	Recept von einem besonderen Elixir	Angelica	In pest	Against plague	INT	RESP
*Angelica* spp.	Apiaceae	HERB	26	Recept von einem besonderen Elixir	Angelica	herrl. Magen-Essenz	Against stomach problems	INT	GAST
*Angelica* spp.	Apiaceae	HERB	26	Recept von einem besonderen Elixir	Angelica	Praeservativ vor alle Gifte	Protection against all poisons	INT	ANTI
*Cinnamomum camphora* (L.) J.Presl	Lauraceae	OIL	26	Recept von einem besonderen Elixir	Campher	In pest	Against plague	INT	NERV
*Cinnamomum camphora* (L.) J.Presl	Lauraceae	OIL	26	Recept von einem besonderen Elixir	Campher	In pest	Against plague	INT	RESP
*Cinnamomum camphora* (L.) J.Presl	Lauraceae	OIL	26	Recept von einem besonderen Elixir	Campher	herrl. Magen-Essenz	Against stomach problems	INT	GAST
*Cinnamomum camphora* (L.) J.Presl	Lauraceae	OIL	26	Recept von einem besonderen Elixir	Campher	Praeservativ vor alle Gifte	Protection against all poisons	INT	ANTI
*Commiphora* spp.	Burseraceae	EXUD	26	Recept von einem besonderen Elixir	Myrrhen	In pest	Against plague	INT	NERV
*Commiphora* spp.	Burseraceae	EXUD	26	Recept von einem besonderen Elixir	Myrrhen	In pest	Against plague	INT	RESP
*Commiphora* spp.	Burseraceae	EXUD	26	Recept von einem besonderen Elixir	Myrrhen	herrl. Magen-Essenz	Against stomach problems	INT	GAST
*Commiphora* spp.	Burseraceae	EXUD	26	Recept von einem besonderen Elixir	Myrrhen	Praeservativ vor alle Gifte	Protection against all poisons	INT	ANTI
*Curcuma zedoaria* (Christm.) Roscoe	Zingiberaceae	SUBT	26	Recept von einem besonderen Elixir	Zittwer	In pest	Against plague	INT	NERV
*Curcuma zedoaria* (Christm.) Roscoe	Zingiberaceae	SUBT	26	Recept von einem besonderen Elixir	Zittwer	In pest	Against plague	INT	RESP
*Curcuma zedoaria* (Christm.) Roscoe	Zingiberaceae	SUBT	26	Recept von einem besonderen Elixir	Zittwer	herrl. Magen-Essenz	Against stomach problems	INT	GAST
*Curcuma zedoaria* (Christm.) Roscoe	Zingiberaceae	SUBT	26	Recept von einem besonderen Elixir	Zittwer	Praeservativ vor alle Gifte	Protection against all poisons	INT	ANTI
*Dictamnus albus* L.	Rutaceae	HERB	26	Recept von einem besonderen Elixir	Weiss Diptam	In pest	Against plague	INT	RESP
*Dictamnus albus* L.	Rutaceae	HERB	26	Recept von einem besonderen Elixir	Weiss Diptam	In pest	Against plague	INT	NERV
*Dictamnus albus* L.	Rutaceae	HERB	26	Recept von einem besonderen Elixir	Weiss Diptam	herrl. Magen-Essenz	Against stomach problems	INT	GAST
*Dictamnus albus* L.	Rutaceae	HERB	26	Recept von einem besonderen Elixir	Weiss Diptam	Praeservativ vor alle Gifte	Protection against all poisons	INT	ANTI
*Gentiana* spp.	Gentianaceae	HERB	26	Recept von einem besonderen Elixir	Entian	In pest	Against plague	INT	NERV
*Gentiana* spp.	Gentianaceae	HERB	26	Recept von einem besonderen Elixir	Entian	In pest	Against plague	INT	RESP
*Gentiana* spp.	Gentianaceae	HERB	26	Recept von einem besonderen Elixir	Entian	herrl. Magen-Essenz	Against stomach problems	INT	GAST
*Gentiana* spp.	Gentianaceae	HERB	26	Recept von einem besonderen Elixir	Entian	Praeservativ vor alle Gifte	Protection against all poisons	INT	ANTI
*Potentilla erecta* (L.) Räusch.	Rosaceae	SUBT	26	Recept von einem besonderen Elixir	Tormentille	In pest	Against plague	INT	NERV
*Potentilla erecta* (L.) Räusch.	Rosaceae	SUBT	26	Recept von einem besonderen Elixir	Tormentille	In pest	Against plague	INT	RESP
*Potentilla erecta* (L.) Räusch.	Rosaceae	SUBT	26	Recept von einem besonderen Elixir	Tormentille	herrl. Magen-Essenz	Against stomach problems	INT	GAST
*Potentilla erecta* (L.) Räusch.	Rosaceae	SUBT	26	Recept von einem besonderen Elixir	Tormentille	Praeservativ vor alle Gifte	Protection against all poisons	INT	ANTI
*Rheum rhabarbarum* L.	Polygonaceae	HERB	26	Recept von einem besonderen Elixir	Rhabarbara	In pest	Against plague	INT	NERV
*Rheum rhabarbarum* L.	Polygonaceae	HERB	26	Recept von einem besonderen Elixir	Rhabarbara	In pest	Against plague	INT	RESP
*Rheum rhabarbarum* L.	Polygonaceae	HERB	26	Recept von einem besonderen Elixir	Rhabarbara	herrl. Magen-Essenz	Against stomach problems	INT	GAST
*Rheum rhabarbarum* L.	Polygonaceae	HERB	26	Recept von einem besonderen Elixir	Rhabarbara	Praeservativ vor alle Gifte	Protection against all poisons	INT	ANTI
–	Mineral	–	27	Krampf-Pulver	Arcanum duplicatum	Krampf	Antispasmodic	INT	MUSK
–	Mineral	–	27	Krampf-Pulver	Antimon daphoreticum	Krampf	Antispasmodic	INT	MUSK
–	Mineral	–	27	Krampf-Pulver	Tartarus vitriolatus	Krampf	Antispasmodic	INT	MUSK
–	Mineral	–	27	Krampf-Pulver	Cinabar antimon	Krampf	Antispasmodic	INT	MUSK
*Cinnamomum camphora* (L.) J.Presl	Lauraceae	OIL	28	Theriac oder Mithridat	Campher	Theriac [as antidote to poisons, especially on viper venom]	Antidote	INT	ANTI
*Juniperus* spp.	Cupressaceae	EXUD	28	Theriac oder Mithridat	Jochandel-Saft	Theriac [as antidote to poisons, especially on viper venom]	Antidote	INT	ANTI
*Laurus nobilis* L.	Lauraceae	HERB	28	Theriac oder Mithridat	Lorbeere	Theriac [as antidote to poisons, especially on viper venom]	Antidote	INT	ANTI
*Piper longum* L.	Piperaceae	SEED	28	Theriac oder Mithridat	langen Pfeffer	Theriac [as antidote to poisons, especially on viper venom]	Antidote	INT	ANTI
*Zingiber officinale* Roscoe	Zingiberaceae	SUBT	28	Theriac oder Mithridat	Ingwer	Theriac [as antidote to poisons, especially on viper venom]	Antidote	INT	ANTI
*Coriandrum sativum* L.	Apiaceae	HERB	29	Aqua Hungarica, Schlagwasser	Coriander	–	–	–	–
*Rosmarinus officinalis* L.	Lamiaceae	HERB	29	Aqua Hungarica, Schlagwasser	Rosmarin	–	–	–	–
*–*	Animal	–	30	Scorpion-Oel	Scorpione	–	–	–	–
*Olea europaea* L.	Oleaceae	EXUD	30	Scorpion-Oel	Baumöl	–	–	–	–
–	Animal	–	31	Sal. volatile-Cornu Cervi, Flüchtig. Hirschhorn-Salz	Cornu Cervi	Pestilenz	Against plague	INT	NERV
–	Animal	–	31	Sal. volatile-Cornu Cervi, Flüchtig. Hirschhorn-Salz	Cornu Cervi	Pestilenz	Against plague	INT	RESP
–	Mineral	–	31	Sal. volatile-Cornu Cervi, Flüchtig. Hirschhorn-Salz	Salz	Pestilenz	Against plague	INT	NERV
–	Mineral	–	31	Sal. volatile-Cornu Cervi, Flüchtig. Hirschhorn-Salz	Salz	Pestilenz	Against plague	INT	RESP
–	Iinctura	–	31	Sal. volatile-Cornu Cervi, Flüchtig. Hirschhorn-Salz	Sp. Vini	Pestilenz	Against plague	INT	NERV
–	Tinctura	–	31	Sal. volatile-Cornu Cervi, Flüchtig. Hirschhorn-Salz	Sp. Vini	Pestilenz	Against plague	INT	RESP
–	Mineral	–	31	Sal. volatile-Cornu Cervi, Flüchtig. Hirschhorn-Salz	Salz	Suchen	Epidemics	INT	–
–	Animal	–	31	Sal. volatile-Cornu Cervi, Flüchtig. Hirschhorn-Salz	Cornu Cervi	Suchen	Epidemics	INT	–
–	Tinctura	–	31	Sal. volatile-Cornu Cervi, Flüchtig. Hirschhorn-Salz	Sp. Vini	Suchen	Epidemics	INT	–
–	Animal	–	31	Sal. volatile-Cornu Cervi, Flüchtig. Hirschhorn-Salz	Cornu Cervi	trefflich Schweissstreibendes Mittel	Excellent diaphoretic	INT	DIAPH
–	Mineral	–	31	Sal. volatile-Cornu Cervi, Flüchtig. Hirschhorn-Salz	Salz	trefflich Schweissstreibendes Mittel	Excellent diaphoretic	INT	DIAPH
–	Tinctura	–	31	Sal. volatile-Cornu Cervi, Flüchtig. Hirschhorn-Salz	Sp. Vini	trefflich Schweissstreibendes Mittel	Excellent diaphoretic	INT	DIAPH
–	Animal	–	31	Sal. volatile-Cornu Cervi, Flüchtig. Hirschhorn-Salz	Cornu Cervi	Fiebern	Fevers	INT	FEV
–	Mineral	–	31	Sal. volatile-Cornu Cervi, Flüchtig. Hirschhorn-Salz	Salz	Fiebern	Fevers	INT	FEV
–	Tinctura	–	31	Sal. volatile-Cornu Cervi, Flüchtig. Hirschhorn-Salz	Sp. Vini	Fiebern	Fevers	INT	FEV
–	Animal	–	31	Sal. volatile-Cornu Cervi, Flüchtig. Hirschhorn-Salz	Cornu Cervi	Hauptschmerzen	Headache	INT	NERV
–	Mineral	–	31	Sal. volatile-Cornu Cervi, Flüchtig. Hirschhorn-Salz	Salz	Hauptschmerzen	Headache	INT	NERV
–	Tinctura	–	31	Sal. volatile-Cornu Cervi, Flüchtig. Hirschhorn-Salz	Sp. Vini	Hauptschmerzen	Headache	INT	NERV
–	Animal	–	31	Sal. volatile-Cornu Cervi, Flüchtig. Hirschhorn-Salz	Cornu Cervi	Steck und Schlag-Flüssen	Prevents stroke	INT	NERV
–	Mineral	–	31	Sal. volatile-Cornu Cervi, Flüchtig. Hirschhorn-Salz	Salz	Steck und Schlag-Flüssen	Prevents stroke	INT	NERV
–	Tinctura	–	31	Sal. volatile-Cornu Cervi, Flüchtig. Hirschhorn-Salz	Sp. Vini	Steck und Schlag-Flüssen	Prevents stroke	INT	NERV
–	Mineral	–	32	Schwarzenberger Heil- und Wundpflaster	Rubrick [as “Rubrica fabrilis (Rötel); bekannt war auch Siegelerde aus Striegau”]	flüssigen alten Schäden	Healing old wounds	EXT	DERM
–	Organic	–	32	Schwarzenberger Heil- und Wundpflaster	Wachs	flüssigen alten Schäden	Healing old wounds	EXT	DERM
–	Mineral	–	32	Schwarzenberger Heil- und Wundpflaster	Rubrick [as “Rubrica fabrilis (Rötel); bekannt war auch Siegelerde aus Striegau”]	in allen hitzigen Schäden	In all types of burns	EXT	DERM
–	Organic	–	32	Schwarzenberger Heil- und Wundpflaster	Wachs	in allen hitzigen Schäden	In all types of burns	EXT	DERM
*Cinnamomum camphora* (L.) J.Presl	Lauraceae	OIL	32	Schwarzenberger Heil- und Wundpflaster	Campher	flüssigen alten Schäden	Healing old wounds	EXT	DERM
*Cinnamomum camphora* (L.) J.Presl	Lauraceae	OIL	32	Schwarzenberger Heil- und Wundpflaster	Campher	in allen hitzigen Schäden	In all types of burns	EXT	DERM
*Olea europaea* L.	Oleaceae	EXUD	32	Schwarzenberger Heil- und Wundpflaster	Baumöl	flüssigen alten Schäden	Healing old wounds	EXT	DERM
*Olea europaea* L.	Oleaceae	EXUD	32	Schwarzenberger Heil- und Wundpflaster	Baumöl	in allen hitzigen Schäden	In all types of burns	EXT	DERM
–	Mineral	–	33	Nürnberger Salben	Rubrick [as “Rubrica fabrilis (Rötel); bekannt war auch Siegelerde aus Striegau”]	flüssigen alten Schäden	Healing old wounds	EXT	DERM
–	Organic	–	33	Nürnberger Salben	Wachs	flüssigen alten Schäden	Healing old wounds	EXT	DERM
*Cinnamomum camphora* (L.) J.Presl	Lauraceae	OIL	33	Nürnberger Salben	Campher	flüssigen alten Schäden	Healing old wounds	EXT	DERM
*Olea europaea* L.	Oleaceae	EXUD	33	Nürnberger Salben	Baumöl	flüssigen alten Schäden	Healing old wounds	EXT	DERM
–	Animal	–	34	Grüne Waldsalbe	Bock-Inselt [as tallow goat]	heilet alle Wunden	Healing all wounds	EXT	DERM
–	Organic	–	34	Grüne Waldsalbe	Wachs	heilet alle Wunden	Healing all wounds	EXT	DERM
–	Organic	–	34	Grüne Waldsalbe	Grünspan	heilet alle Wunden	Healing all wounds	EXT	DERM
?	?	EXUD	34	Grüne Waldsalbe	Hartz [as resin]	heilet alle Wunden	Healing all wounds	EXT	DERM
?	?	EXUD	34	Grüne Waldsalbe	Terpentin	heilet alle Wunden	Healing all wounds	EXT	DERM
–	Inorganic	–	35	Oleum Montis, Berg-Oel	Balsam Sulphuris	–	–	–	–
–	Animal	–	35	Oleum Montis, Berg-Oel	Oleum Cornu Cervi	–	–	–	–
?	?	EXUD	35	Oleum Montis, Berg-Oel	Oleum Therebinth	–	–	–	–
*Linum usitatissimum* L.	Linaceae	OIL	35	Oleum Montis, Berg-Oel	Oleum Lini	–	–	–	–
–	Animal	–	36	Franzosen-Oel, Oleum Cuajaci	Cornu Cervi	Franzosen	Syphilis	INT	GYN
–	Animal	–	36	Franzosen-Oel, Oleum Cuajaci	Cornu Cervi	Franzosen	Syphilis	INT	ANDR
*Guaiacum officinale* L.	Zygophyllaceae	OIL	36	Franzosen-Oel, Oleum Cuajaci	Oleum Guajaci	Franzosen	Syphilis	INT	ANDR
*Guaiacum officinale* L.	Zygophyllaceae	OIL	36	Franzosen-Oel, Oleum Cuajaci	Oleum Guajaci	Franzosen	Syphilis	INT	GYN
*Linum usitatissimum* L.	Linaceae	OIL	36	Franzosen-Oel, Oleum Cuajaci	Oleum Lini	Franzosen	Syphilis	INT	ANDR
*Linum usitatissimum* L.	Linaceae	OIL	36	Franzosen-Oel, Oleum Cuajaci	Oleum Lini	Franzosen	Syphilis	INT	GYN
*Achillea* spp.	Asteraceae	HERB	37	guthe Kräuter-Thee	Schaafgarben	Blutreinigung	Blood purification	INT	CARD
*Achillea* spp.	Asteraceae	HERB	37	guthe Kräuter-Thee	Schaafgarben	Lungenreinigung	Cleansing the lungs	INT	RESP
*Achillea* spp.	Asteraceae	HERB	37	guthe Kräuter-Thee	Schaafgarben	Brustreinigung	Cleansing the upper respiratory tract	INT	RESP
*Bellis perennis* L.	Asteraceae	FLOW	37	guthe Kräuter-Thee	Gänseblümel	Blutreinigung	Blood purification	INT	CARD
*Bellis perennis* L.	Asteraceae	FLOW	37	guthe Kräuter-Thee	Gänseblümel	Lungenreinigung	Cleansing the lungs	INT	RESP
*Bellis perennis* L.	Asteraceae	FLOW	37	guthe Kräuter-Thee	Gänseblümel	Brustreinigung	Cleansing the upper respiratory tract	INT	RESP
*Stachys officinalis* (L.) Trevis = *Betonica officinalis* L.	Lamiaceae	FLOW	37	guthe Kräuter-Thee	Betonien-Blumen	Blutreinigung	Blood purification	INT	CARD
*Stachys officinalis* (L.) Trevis = *Betonica officinalis* L.	Lamiaceae	FLOW	37	guthe Kräuter-Thee	Betonien-Blumen	Lungenreinigung	Cleansing the lungs	INT	RESP
*Stachys officinalis* (L.) Trevis = *Betonica officinalis* L.	Lamiaceae	FLOW	37	guthe Kräuter-Thee	Betonien-Blumen	Brustreinigung	Cleansing the upper respiratory tract	INT	RESP
*Cyanus segetum* Hill. = *Centaurea cyanus* L.	Asteraceae	HERB	37	guthe Kräuter-Thee	Kornblumen	Blutreinigung	Blood purification	INT	CARD
*Cyanus segetum* Hill. = *Centaurea cyanus* L.	Asteraceae	HERB	37	guthe Kräuter-Thee	Kornblumen	Lungenreinigung	Cleansing the lungs	INT	RESP
*Cyanus segetum* Hill. = *Centaurea cyanus* L.	Asteraceae	HERB	37	guthe Kräuter-Thee	Kornblumen	Brustreinigung	Cleansing the upper respiratory tract	INT	RESP
*Citrus* ×*aurantium* L.	Rutaceae	FRU	37	guthe Kräuter-Thee	Pommeranzschaalen	Blutreinigung	Blood purification	INT	CARD
*Citrus* ×*aurantium* L.	Rutaceae	FRU	37	guthe Kräuter-Thee	Pommeranzschaalen	Lungenreinigung	Cleansing the lungs	INT	RESP
*Citrus* ×*aurantium* L.	Rutaceae	FRU	37	guthe Kräuter-Thee	Pommeranzschaalen	Brustreinigung	Cleansing the upper respiratory tract	INT	RESP
*Citrus limon* (L.) Osbeck	Rutaceae	FRU	37	guthe Kräuter-Thee	Citron	Blutreinigung	Blood purification	INT	CARD
*Citrus limon* (L.) Osbeck	Rutaceae	FRU	37	guthe Kräuter-Thee	Citron	Lungenreinigung	Cleansing the lungs	INT	RESP
*Citrus limon* (L.) Osbeck	Rutaceae	FRU	37	guthe Kräuter-Thee	Citron	Brustreinigung	Cleansing the upper respiratory tract	INT	RESP
*Coriandrum sativum* L.	Apiaceae	HERB	37	guthe Kräuter-Thee	Coriander	Blutreinigung	Blood purification	INT	CARD
*Coriandrum sativum* L.	Apiaceae	HERB	37	guthe Kräuter-Thee	Coriander	Lungenreinigung	Cleansing the lungs	INT	RESP
*Coriandrum sativum* L.	Apiaceae	HERB	37	guthe Kräuter-Thee	Coriander	Brustreinigung	Cleansing the upper respiratory tract	INT	RESP
*Echium vulgare* L.	Boraginaceae	LEAF	37	guthe Kräuter-Thee	Natterblätter	Blutreinigung	Blood purification	INT	CARD
*Echium vulgare* L.	Boraginaceae	LEAF	37	guthe Kräuter-Thee	Natterblätter	Lungenreinigung	Cleansing the lungs	INT	RESP
*Echium vulgare* L.	Boraginaceae	LEAF	37	guthe Kräuter-Thee	Natterblätter	Brustreinigung	Cleansing the upper respiratory tract	INT	RESP
*Glycyrrhiza glabra* L.	Fabaceae	HERB	37	guthe Kräuter-Thee	Süsseholz	Blutreinigung	Blood purification	INT	CARD
*Glycyrrhiza glabra* L.	Fabaceae	HERB	37	guthe Kräuter-Thee	Süsseholz	Lungenreinigung	Cleansing the lungs	INT	RESP
*Glycyrrhiza glabra* L.	Fabaceae	HERB	37	guthe Kräuter-Thee	Süsseholz	Brustreinigung	Cleansing the upper respiratory tract	INT	RESP
*Guaiacum sanctum* L. or *G. officinale* L.	Zygophyllaceae	WOOD	37	guthe Kräuter-Thee	Lignum Sanctum	Blutreinigung	Blood purification	INT	CARD
*Guaiacum sanctum* L. or *G. officinale* L.	Zygophyllaceae	WOOD	37	guthe Kräuter-Thee	Lignum Sanctum	Lungenreinigung	Cleansing the lungs	INT	RESP
*Guaiacum sanctum* L. or *G. officinale* L.	Zygophyllaceae	WOOD	37	guthe Kräuter-Thee	Lignum Sanctum	Brustreinigung	Cleansing the upper respiratory tract	INT	RESP
*Hedysarum* spp.	Fabaceae	HERB	37	guthe Kräuter-Thee	Süssen Klee	Blutreinigung	Blood purification	INT	CARD
*Hedysarum* spp.	Fabaceae	HERB	37	guthe Kräuter-Thee	Süssen Klee	Lungenreinigung	Cleansing the lungs	INT	RESP
*Hedysarum* spp.	Fabaceae	HERB	37	guthe Kräuter-Thee	Süssen Klee	Brustreinigung	Cleansing the upper respiratory tract	INT	RESP
*Matricaria* spp.	Asteraceae	HERB	37	guthe Kräuter-Thee	Camillen	Blutreinigung	Blood purification	INT	CARD
*Matricaria* spp.	Asteraceae	HERB	37	guthe Kräuter-Thee	Camillen	Lungenreinigung	Cleansing the lungs	INT	RESP
*Matricaria* spp.	Asteraceae	HERB	37	guthe Kräuter-Thee	Camillen	Brustreinigung	Cleansing the upper respiratory tract	INT	RESP
*Ononis spinosa* L.	Fabaceae	HERB	37	guthe Kräuter-Thee	Steinwurzel	Blutreinigung	Blood purification	INT	CARD
*Ononis spinosa* L.	Fabaceae	HERB	37	guthe Kräuter-Thee	Steinwurzel	Lungenreinigung	Cleansing the lungs	INT	RESP
*Ononis spinosa* L.	Fabaceae	HERB	37	guthe Kräuter-Thee	Steinwurzel	Brustreinigung	Cleansing the upper respiratory tract	INT	RESP
*Pulicaria vulgaris* Gaertn.	Asteraceae	HERB	37	guthe Kräuter-Thee	Cristinel-Kraut	Blutreinigung	Blood purification	INT	CARD
*Pulicaria vulgaris* Gaertn.	Asteraceae	HERB	37	guthe Kräuter-Thee	Cristinel-Kraut	Lungenreinigung	Cleansing the lungs	INT	RESP
*Pulicaria vulgaris* Gaertn.	Asteraceae	HERB	37	guthe Kräuter-Thee	Cristinel-Kraut	Brustreinigung	Cleansing the upper respiratory tract	INT	RESP
*Pyrus* sp.	Rosaceae	FRU	37	guthe Kräuter-Thee	Kragel [as the common name of the old pear variety Kragel Birne]	Blutreinigung	Blood purification	INT	CARD
*Pyrus* sp.	Rosaceae	FRU	37	guthe Kräuter-Thee	Kragel [as the common name of the old pear variety Kragel Birne]	Lungenreinigung	Cleansing the lungs	INT	RESP
*Pyrus* sp.	Rosaceae	FRU	37	guthe Kräuter-Thee	Kragel [as the common name of the old pear variety Kragel Birne]	Brustreinigung	cleansing the upper respiratory tract	INT	RESP
*Rosa* spp.	Rosaceae	FLOW	37	guthe Kräuter-Thee	Rosenblätter	Blutreinigung	Blood purification	INT	CARD
*Rosa* spp.	Rosaceae	FLOW	37	guthe Kräuter-Thee	Rosenblätter	Lungenreinigung	Cleansing the lungs	INT	RESP
*Rosa* spp.	Rosaceae	FLOW	37	guthe Kräuter-Thee	Rosenblätter	Brustreinigung	Cleansing the upper respiratory tract	INT	RESP
*Sassafras* spp.	Lauraceae	HERB	37	guthe Kräuter-Thee	Sassafras	Blutreinigung	Blood purification	INT	CARD
*Sassafras* spp.	Lauraceae	HERB	37	guthe Kräuter-Thee	Sassafras	Lungenreinigung	Cleansing the lungs	INT	RESP
*Sassafras* spp.	Lauraceae	HERB	37	guthe Kräuter-Thee	Sassafras	Brustreinigung	Cleansing the upper respiratory tract	INT	RESP
*Scabiosa* spp.	Dipsacaceae	HERB	37	guthe Kräuter-Thee	Scabiosen	Blutreinigung	Blood purification	INT	CARD
*Scabiosa* spp.	Dipsacaceae	HERB	37	guthe Kräuter-Thee	Scabiosen	Lungenreinigung	Cleansing the lungs	INT	RESP
*Scabiosa* spp.	Dipsacaceae	HERB	37	guthe Kräuter-Thee	Scabiosen	Brustreinigung	Cleansing the upper respiratory tract	INT	RESP
*Silene baccifera* (L.) Roth = *Cucubalus baccifer* L.	Caryophyllaceae	HERB	37	guthe Kräuter-Thee	Taubenkropf	Blutreinigung	Blood purification	INT	CARD
*Silene baccifera* (L.) Roth = *Cucubalus baccifer* L.	Caryophyllaceae	HERB	37	guthe Kräuter-Thee	Taubenkropf	Lungenreinigung	Cleansing the lungs	INT	RESP
*Silene baccifera* (L.) Roth = *Cucubalus baccifer* L.	Caryophyllaceae	HERB	37	guthe Kräuter-Thee	Taubenkropf	Brustreinigung	Cleansing the upper respiratory tract	INT	RESP
*Vaccinium vitis*–*idaea* L.	Ericaceae	HERB	37	guthe Kräuter-Thee	Preusselbeeren-Kraut	Blutreinigung	Blood purification	INT	CARD
*Vaccinium vitis*–*idaea* L.	Ericaceae	HERB	37	guthe Kräuter-Thee	Preusselbeeren-Kraut	Lungenreinigung	Cleansing the lungs	INT	RESP
*Vaccinium vitis*–*idaea* L.	Ericaceae	HERB	37	guthe Kräuter-Thee	Preusselbeeren-Kraut	Brustreinigung	Cleansing the upper respiratory tract	INT	RESP
*Veronica* spp.	Plantaginaceae	HERB	37	guthe Kräuter-Thee	Ehrenpreis	Blutreinigung	Blood purification	INT	CARD
*Veronica* spp.	Plantaginaceae	HERB	37	guthe Kräuter-Thee	Ehrenpreis	Lungenreinigung	Cleansing the lungs	INT	RESP
*Veronica* spp.	Plantaginaceae	HERB	37	guthe Kräuter-Thee	Ehrenpreis	Brustreinigung	Cleansing the upper respiratory tract	INT	RESP
–	Tinctura	–	38	Tinctur Bezoardica F. Grossmann Krummhübler Art.	Sp. Vini	–	–	INT	–
*Actaea racemosa* L. = *Cimicifuga racemosa* Nutt.	Ranunculaceae	SUBT	38	Tinctur Bezoardica F. Grossmann Krummhübler Art.	Radix Serpentariae	–	–	INT	–
*Carlina acaulis* L.	Asteraceae	SUBT	38	Tinctur Bezoardica F. Grossmann Krummhübler Art.	Radix Carlinae	–	–	INT	–
*Cinnamomum camphora* (L.) J.Presl	Lauraceae	OIL	38	Tinctur Bezoardica F. Grossmann Krummhübler Art.	Campher	–	–	INT	–
*Curcuma zedoaria* (Christm.) Roscoe	Zingiberaceae	SUBT	38	Tinctur Bezoardica F. Grossmann Krummhübler Art.	Radix Zedoar	–	–	INT	–
*Dictamnus albus* L.	Rutaceae	HERB	38	Tinctur Bezoardica F. Grossmann Krummhübler Art.	Radix Diptam alb.	–	–	INT	–
*Petroselinum crispum* (Mill.) Fuss.	Apiaceae	SUBT	38	Tinctur Bezoardica F. Grossmann Krummhübler Art.	Radix Petros[elini]	–	–	INT	–
*Peucedanum ostruthium* (L.) W.D.J. Koch = *Imperatoria ostruthium* L.	Apiaceae	SUBT	38	Tinctur Bezoardica F. Grossmann Krummhübler Art.	Radix Imperat.	–	–	INT	–
*Pterocarpus santalinus* L. fil.	Fabaceae	HERB	38	Tinctur Bezoardica F. Grossmann Krummhübler Art.	rothen Sandel	–	–	INT	–
–	Inorganic	–	39	Tinctur Lunae, Tinktur von Silber	Silber	in bösen Wesen	Against postpartum depression	INT	NERV
–	Organic	–	39	Tinctur Lunae, Tinktur von Silber	Urine	in bösen Wesen	Against postpartum depression	INT	NERV
–	Tinctura	–	39	Tinctur Lunae, Tinktur von Silber	Sp. Vini	in bösen Wesen	Against postpartum depression	INT	NERV
–	Inorganic	–	39	Tinctur Lunae, Tinktur von Silber	Silber	Haupt-Krankheiten	Brain disease	INT	NERV
–	Organic	–	39	Tinctur Lunae, Tinktur von Silber	Urine	Haupt-Krankheiten	Brain disease	INT	NERV
–	Tinctura	–	39	Tinctur Lunae, Tinktur von Silber	Sp. Vini	Haupt-Krankheiten	Brain disease	INT	NERV
–	Organic	–	40	Sp. Tartari, Weinstein-Geist	Weinstein (tartar)	in der Gicht	Against gout	INT	MUSK
–	Organic	–	40	Sp. Tartari, Weinstein-Geist	Weinstein (tartar)	bey lahmen Gliedern	Against lame limbs	INT	MUSK
–	Organic	–	40	Sp. Tartari, Weinstein-Geist	Weinstein (tartar)	in Lähmung	Against paralysis	INT	NERV
–	Organic	–	40	Sp. Tartari, Weinstein-Geist	Weinstein (tartar)	in Scharbock	Against scurvy	INT	MUSK
–	Organic	–	40	Sp. Tartari, Weinstein-Geist	Weinstein (tartar)	Wassersucht	Oedema	INT	CARD
–	Organic	–	40	Sp. Tartari, Weinstein-Geist	Weinstein (tartar)	Räudigkeit der Haut	Erythema	INT	DERM
–	Organic	–	40	Sp. Tartari, Weinstein-Geist	Weinstein (tartar)	eröfnet Verstopfung des Eingeweides	Removes intestinal obstruction	INT	GAST
–	Organic	–	40	Sp. Tartari, Weinstein-Geist	Weinstein (tartar)	Krätze	Scabies	INT	DERM
–	Organic	–	40	Sp. Tartari, Weinstein-Geist	Weinstein (tartar)	Franzosen	Syphilis	INT	ANDR
–	Organic	–	40	Sp. Tartari, Weinstein-Geist	Weinstein (tartar)	Franzosen	Syphilis	INT	GYN
–	Organic	–	40	Sp. Tartari, Weinstein-Geist	Weinstein (tartar)	Windsucht	Tympanites	INT	GAST
–	Elixir	–	41	Liquor anod Michaeli	rothen Schlagwasser oder Englischen Balsam	–	–	–	–
–	Inorganic	–	41	Liquor anod Michaeli	Sp. Nitri dulcis	–	–	–	–
–	Inorganic	–	42	Sp. Salammoniaci anisat. Salmiac-Geist mit Anis	Kalck	Magen	Against stomach problems	INT	GAST
–	Inorganic	–	42	Sp. Salammoniaci anisat. Salmiac-Geist mit Anis	Kalck	Nieren	For the kidneys	INT	URO
–	Inorganic	–	42	Sp. Salammoniaci anisat. Salmiac-Geist mit Anis	Kalck	stärkt die Brust	Strengthens the breast	INT	RESP
*Glycyrrhiza glabra* L. (as salty liquorice)	Fabaceae	SUBT	42	Sp. Salammoniaci anisat. Salmiac-Geist mit Anis	Salmiac	Magen	Against stomach problems	INT	GAST
*Glycyrrhiza glabra* L. (as salty liquorice)	Fabaceae	SUBT	42	Sp. Salammoniaci anisat. Salmiac-Geist mit Anis	Salmiac	Nieren	For the kidneys	INT	URO
*Glycyrrhiza glabra* L. (as salty liquorice)	Fabaceae	SUBT	42	Sp. Salammoniaci anisat. Salmiac-Geist mit Anis	Salmiac	stärkt die Brust	Strengthens the breast	INT	RESP
*Pimpinella anisum* L.	Apiaceae	HERB	42	Sp. Salammoniaci anisat. Salmiac-Geist mit Anis	Anis	Magen	Against stomach problems	INT	GAST
*Pimpinella anisum* L.	Apiaceae	HERB	42	Sp. Salammoniaci anisat. Salmiac-Geist mit Anis	Anis	Nieren	For the kidneys	INT	URO
*Pimpinella anisum* L.	Apiaceae	HERB	42	Sp. Salammoniaci anisat. Salmiac-Geist mit Anis	Anis	stärkt die Brust	Strengthens the breast	INT	RESP
*–*	Tinctura	–	43	Unächt. Recept zur Ess. dulcis	Spiritus abgezogen von Englisch Balsam oder vom rothen Schlagwasser	–	–	–	–
*Pterocarpus santalinus* L. fil.	Fabaceae	HERB	43	Unächt. Recept zur Ess. dulcis	rothen Sandel	–	–	–	–
–	Tinctura	–	44	Tinct. Benzoes	Sp. Vini	vor die Brust	For the breast	INT	RESP
–	Tinctura	–	44	Tinct. Benzoes	Sp. Vini	vor die Brust	For the breast	EXT	OTH
*Styrax* spp.	Styracaceae	EXUD	44	Tinct. Benzoes	Gummi Benzoe	vor die Brust	For the breast	INT	RESP
*Styrax* spp.	Styracaceae	EXUD	44	Tinct. Benzoes	Gummi Benzoe	vor die Brust	For the breast	EXT	OTH
*Rosa* spp.	Rosaceae	FLOW	45	Tinctura Rosarum, Rosen-Tinctur	Rosenblätter	kühlet und stärket das Herz	Strengthens the heart	INT	CARD
*Rosa* spp.	Rosaceae	FLOW	45	Tinctura Rosarum, Rosen-Tinctur	Rosenblätter	stärket die Leber	Strengthens the liver	INT	GAST
*Rosa* spp.	Rosaceae	FLOW	45	Tinctura Rosarum, Rosen-Tinctur	Rosenblätter	stärket den Magen	Strengthens the stomach	INT	GAST
*Gentiana* spp.	Gentianaceae	SUBT	46	Ess. Gentiana, Enzian-Wurzel-Essenz	Enzian-Wurzel	allen 3 und 4 tägigten Fiebern	In all 3 and 4 days of fever	INT	FEV
*Gentiana* spp.	Gentianaceae	SUBT	46	Ess. Gentiana, Enzian-Wurzel-Essenz	Enzian-Wurzel	in Schwachheit des Magens	In weakness of the stomach	INT	GAST

### Therapeutic effects of medicinal plants in traditional and modern medicine

It is estimated that over 50% of the available drugs are currently somehow derived from medicinal plants [67, 68]. Herbal medicine (phytotherapy) is widely being used across the world on a constantly growing basis. Plant drug application is based on the experiences of traditional medicine or on new scientific research and experimental results, i.e. conventional medicine. Many medicinal plants are applied through self-medication or at the recommendation of a physician or pharmacist [69]. Phytotherapy is among the major “complementary” treatments in current use by doctors and other therapists throughout Europe [70]. Contemporary European use and trade in medicinal and aromatic plants are extensive, with eight countries (Germany, Spain, France, the Netherlands, Italy, the UK, the Russian Federation (not disaggregated by Russia-in-Europe) and Poland) being the top 20 global importers by volume of pharmaceutical plants. The top six exporters of these plants in Europe include Germany, Poland, Spain, Bulgaria, Albania and France [71, 72]. A large part of modern drugs has its roots in ancient traditions. Until today, ancient scripts have exerted a strong influence on the use of herbal medicine, and the repeated empirical testing and scientific study of health care claims guide and shape the selection of efficacious treatments and evidence-based herbal medicine [73].

Medicinal plants used by herbalists from Krummhübel were remedies for multiple ailments. The taxa that achieved the highest use or were recognised as the most versatile remedies with multiple pharmacological indications were *Aloë* spp., *Copaifera officinalis* L., *Guaiacum officinale* L., *Commiphora* spp. and *Crocus* (probably) *sativus* L. Comparison of the uses of the plants considered with their contemporary use, described in publications involved with herbal medicine and pharmacognosy (e.g. [36, 74–77]), showed some novelties.

The most frequently mentioned properties of *Aloë* spp. are gastrointestinal activities, hepato-protective properties and beneficial effects against skin problems such as wounds, injuries and infective diseases in both the Islamic traditional medicine [78] and in modern medicine [36, 74, 76, 77]. According to Krummhübel herbalists, it has also antihelminthic properties and can be used as a remedy for the treatment of scurvy.

Copaiba (*Copaifera* sp.) has a wide range of ethnopharmacological indications, including the treatment of the following: cystitis, urinary incontinence, gonorrhoea and syphilis; respiratory ailments including bronchitis, strep throat, haemoptysis, pneumonia and sinusitis; infections in the skin and mucosa such as dermatitis, eczema, psoriasis and wounds; ulcers and lesions of the uterus; leishmaniasis and leucorrhea; anaemia; headaches; and snake bites. It is also used for its aphrodisiac, stimulant, anti-inflammatory, antiseptic, anti-tetanus, antirheumatic, antiherpetic, anthelminthic, anticancer, antitumour (prostate tumours) and antiparalytic properties ([63] and references cited herein). Many of these indications were also mentioned by Krummhübel herbalists and are recognised by modern medicine [74, 76, 77].

Guaiacum (*Guaiacum officinale* L.) is stated to possess antirheumatic, anti-inflammatory, diuretic, mild laxative and diaphoretic properties [74, 76, 77]. Traditionally, it has been used for subacute rheumatism, also in syphilitic and gouty affections, and specifically for chronic rheumatism and rheumatoid arthritis [75]. Additional medicinal uses mentioned by Krummhübel herbalists include the treatment of oedema and scabies as well as blood purification.

Myrrh is a sap-like substance (resin) that is released from cuts in the bark of trees belonging to the genus *Commiphora*. Myrrh has antimicrobial, astringent, carminative, expectorant, anticatarrhal, antiseptic and vulnerary properties. Traditionally, it has been used for aphthous ulcers, pharyngitis, respiratory catarrhs, common cold, furunculosis, wounds and abrasions, specifically for mouth ulcers, gingivitis and pharyngitis [75]. It is unknown which *Commiphora* species was used by Krummhübel herbalists, but they recommended it, among others, as a remedy for scurvy and plague as well as to stimulate appetite.

*Crocus sativus* L., commonly known as saffron, is used in folk medicine as an antispasmodic, eupeptic, gingival sedative, anticatarrhal, nerve sedative, carminative, diaphoretic, expectorant, stimulant, stomachic, aphrodisiac and emmenagogue. Furthermore, modern pharmacological studies have demonstrated that saffron extract or its active constituents have antitumor effects, radical scavenger properties and hypolipemic effects [75]. Krummhübel herbalists additionally used this plant in their medical mixtures as remedies for scurvy, any injuries and to support postpartum recovery.

Since time immemorial, people have tried to find medications to alleviate pain and to cure various diseases. In every period, every successive century from the development of humankind and advanced civilisations, the healing properties of certain medicinal plants were identified, recorded and passed on to successive generations. The benefits of one society were conveyed to another, which upgraded the old properties and discovered new ones, until the present days. The continuous and perpetual interest of people in medicinal plants has led to today’s modern and sophisticated fashion of their processing and usage [69].

## Conclusions

This paper presents a data mining approach and a survey of the herbal drugs contained in Reitzig. Our study revealed that many plants used in medical treatments by Krummhübel herbalists were also known in other regions between the sixteenth and twentieth centuries. The medicinal plants documented in all ethnobotanical studies considered include *Angelica* ssp., *Carlina acaulis* L., *Gentiana* spp., *Juniperus* spp., *Rosa* spp. and *Veronica* spp. However, eight, mainly exotic plants, were exclusive in therapeutic mixtures of Krummhübel herbalists. They encompass *Copaifera officinalis* L., *Drimys winteri* J.R. Forst. & G. Forst., *Hedysarum* spp., *Myristica fragrans* Houtt., *Piper longum* L., *Silene baccifera* (L.) Roth and *Syzygium aromaticum* (L.) Merr. & L.M. Perry. Although these taxa originate from various parts of the world, they were quite frequently used in several remedies by Krummhübel herbalists and are still important herbs in modern phytotherapy. Besides, the preserved recipes of Krummhübel herbalists also cover animal, fungus and mineral formulations and other organic and inorganic ones. Comparing such old data with contemporary herbal medicine and phytotherapy might enhance our understanding of modern practices and help to document the tradition of use, which is required for the regulatory approval of new herbal drugs. We showed that therapeutic effects of medicinal plants used by Krummhübel herbalists in traditional and modern medicine are mainly congruent, but there are also some novelties.

Currently, based on the achievements of Krummhübel herbalists, it seems to be important to attempt to reproduce therapeutic mixtures from the preserved recipes. This would provide an opportunity to learn more about the real effects of the former medicines and their therapeutic activities. The obtained data can also be used in the search for new drugs.

## Additional file

Additional file 1: A full dataset of the recorded plant taxa, plant parts and other constituents used, as well as the therapeutic uses. (XLSX 37 kb)
